# Synthetic microbiota for microplastic degradation modulates rhizosphere fungal diversity and metabolic function in highland barley

**DOI:** 10.3389/fmicb.2025.1711544

**Published:** 2025-12-08

**Authors:** Yue Deng, Peng Xiang, Mei Zhang, Shouqin Wang, Xudong Zhou, Jincheng Liu, Qiang Li, Guiqiang He

**Affiliations:** 1 Engineering Research Center of Biomass Materials, Ministry of Education, College of Life Sciences and Agri-forestry, Southwest University of Science and Technology, Mianyang, Sichuan, China; 2 Luzhou Vocational and Technical College, Luzhou, Sichuan, China; 3 College of Food and Biological Engineering, Chengdu University, Chengdu, Sichuan, China

**Keywords:** polystyrene microplastics, synthetic microbiota, rhizosphere fungi, bioremediation, fungal community diversity

## Abstract

Microplastic (MPs) pollution is a growing concern for agricultural sustainability and crop nutritional quality. This study examined the individual and combined effects of polystyrene MPs (varying in particle size: <1 mm and 1–5 mm; and concentration: 1, 10, and 50 g/m^2^) and a synthetic microbiota consortium tailored for MP degradation (MPDSM) on the grain nutritional profile and rhizosphere fungal communities of highland barley. Application of MPDSM significantly enhanced MPs degradation, achieving a weight loss of 19.9% for large particles and 7.4% for small particles. MPs contamination reduced zinc content in grains, while particle size differentially modulated phytochemical composition: larger MPs increased flavonoid levels, whereas smaller MPs elevated polyphenol and vitamin E content. Notably, MPDSM treatment improved key nutritional indices, such as fat and vitamin C content. Moreover, the α-diversity of rhizosphere fungi increased under all treatments except under medium-concentration large MPs. The synthetic microbiota specifically enriched fungal diversity and drove community differentiation. FUNGuild analysis indicated a significant functional shift toward a Fungal_Parasite-Undefined_Saprotroph profile. These results demonstrate the potential of tailored synthetic microbiota to mitigate microplastic pollution in agroecosystems via remodeling the rhizosphere fungal community and its metabolic functions, presenting a promising bioremediation strategy for contaminated agricultural soils.

## 1 Introduction

Microplastics (MPs), particles smaller than 5 mm, are produced when plastic waste is broken down through physical, chemical, biological, and other forms of degradation (Roager and Sonnenschein, 2019; Xiang et al., 2023b). MPs are emerging organic pollutants that have been confirmed to be more harmful than plastics (Roager and Sonnenschein, 2019). In agricultural ecosystems, plastic film mulching and wastewater irrigation are significant pathways for microplastic input. When microplastics enter the soil, they become incorporated into the soil matrix through tillage, bioturbation, and wet-dry cycles (Steinmetz et al., 2016; Gao et al., 2019). This incorporation can alter soil bulk density, porosity, water-holding capacity, pH, and nutrient content. Such changes in physicochemical properties can further negatively impact microbial community diversity and crop growth (De Souza Machado et al., 2019; Du and Wang, 2021; Leifheit et al., 2021). Consequently, microplastic pollution in agricultural systems has become a critical scientific concern in the fields of environment and ecology (Horton et al., 2017; Liu et al., 2018).

Highland barley, China's smallest starchy crop and largest coarse grain (Guo et al., 2020), boasts beneficial dietary qualities such as low fat and sugar, high fiber, and notably high β-glucan levels Moreover, it is a source of Vitamin E, which is known to be an important antioxidant (Do et al., 2015). Thus, it is important to enhance the nutritional value of highland barley. The nutrient content and mineral elements of green barley are affected by environmental conditions (Zhang et al., 2021b). Araya's research reveals that the use of plastic film mulching significantly enhances the yield of the highland barley (Araya et al., 2016). While mulching has some economic benefits, it can also be damaged due to the plastic residues left in the soil after harvest (Brodhagen et al., 2017). However, to date, the effect of plastic film mulch on the nutrient and mineral composition of crops remains unknown.

Microbes have been shown to influence the growth of plants by secreting compounds such as auxins, and can also affect crop yield and quality through nutrient mobilization and transfer (Keswani et al., 2022). The rhizosphere microbiome is therefore seen as a major factor in plant health and productivity. MPs are argued to nourish soil microbes, influencing the soil ecosystem ecology. Fungi, which are some of the most abundant organisms in the soil, are also seen as a key element of soil biodiversity and metabolic capacity (Temporiti et al., 2022). As one of the most abundant taxa in soil, fungi play a critical role in soil ecosystem cycling. Numerous studies have indicated that microplastics can affect the structure of soil bacterial communities by acting as a substrate or carbon source (Xiang et al., 2023a; Zhu et al., 2022). However, research on how microplastics influence the structure and function of rhizosphere fungal communities, which are closely associated with crop health, remains insufficient. This knowledge gap hampers a comprehensive understanding of the impact of microplastics on soil ecosystem functionality (Shirin et al., 2024).

Based on microbial remediation technologies, particularly the use of synthetic microbial communities (SynComs), is considered a promising strategy for pollutant control. Synthetic microbial communities achieve efficient degradation of complex pollutants through interspecies collaboration and are expected to enhance their colonization resistance and stress tolerance in complex soil environments. Although numerous microorganisms capable of degrading microplastics have been isolated, constructing specific degrader assemblages into synthetic communities and investigating their effectiveness in degrading microplastics under field conditions, along with their comprehensive impact on the rhizosphere microecology and crop nutrition enhancement, remains a critical and underexplored area in current research.

The objective of this study was to systematically evaluate the combined effects of polystyrene-MPs, with varying particle sizes (< 1 mm and 1–5 mm) and concentrations (1, 10, and 50 g/m^2^), and a microplastic-degrading synthetic microbiota (MPDSM) on the grain nutritional profile of highland barley (*Hordeum vulgare* L.) and the composition and function of the fungal community in its rhizosphere soil. This investigation aims to clarify the impact mechanisms of MPs with different properties on crop nutrition and root-associated microbiota, while assessing the potential of MPDSM inoculation for remediation. By applying MPDSM in field trials and analyzing the coupled responses of crop nutrition and rhizosphere microecology, this study provides systematic insights into the ecological effects of MPs and novel strategies for their bioremediation.

## 2 Materials and methodology

### 2.1 Microplastics and bacteria

PS-MPs were procured from Zhonglian Plastics Technology Co., Ltd. (Guangdong, China). MPs were sterilized by UV irradiation. The microplastic-degrading synthetic microbiota (MPDSM) consisted of three bacterial species previously identified as capable of degrading PS-MPs: *Stenotrophomonas maltophilia, Bacillus velezensis*, and *Acinetobacter radioresistens* (Xiang et al., 2023c). In our prior laboratory degradation experiments, a 1:1:1 mixture of the three strains was found to yield the highest synergistic degradation efficiency of PS-MPs (17.0%) after 60 days, surpassing the performance of any single strain (ranging from 4.1% to 16.7%) (Xiang et al., 2023c). In this experiment, it was important to ensure that at least one of the strains of the added synthetic flora survived and acted as a degrading agent, taking into account the influence of the indigenous bacteria. Therefore, all three strains were cultured in LB medium at 30 °C and 110 rpm with an absorbance of 0.80 ± 0.05 and a survival count of 2 × 10^8^ CFU/mL measured at 600 nm. MPDSM was obtained by mixing the three strains in a 1:1:1 ratio. Through the deposit numbers OQ927056, OQ927057, and OQ927058 at NCBI GenBank, these cultures are cared for at the Chengdu University Microbial Conservation Center.

### 2.2 Experimental design

Chengdu University supplied the CDU-66 Highland barley seeds. The operations spanned the season from September 2023 to March 2024 at the Jianyang, Sichuan location (Latitude: 30°0′, Longitude: 104°5′). No evidence of MPs was found in the initial soil evaluation in the trial area. Predominantly, the soil was made up of both purple and alluvial soil, making it well-suited for farming. The mean experimental temperature was 14 °C, with 874 mm of rainfall, 1,250 h of sunlight, and a frost-free span of 311 days, indicating generally mild weather conditions. Highland barley seeds, meticulously chosen for their quality and bulkiness, underwent a 30-min pre-wash in a 2% sodium hypochlorite solution, followed by a rinse in deionized water and a 6-hour soaking period. The experiment was conducted using uniform seeding. Before experimentation, MPs underwent UV exposure to simulate the natural environment. After seedling, dig the topsoil with a shovel to ensure that the MPs reach the roots of the plants. Grouped into six, differentiated by size and concentration: Large, low-concentration (1–5 mm, 1 g/m^2^), large, medium-concentration (1–5 mm, 10 g/m^2^), large, high-concentration (1–5 mm, 50 g/m^2^), small, low-concentration (< 1 mm, 1 g/m^2^), small, medium-concentration (< 1 mm, 10 g/m^2^), and small, high-concentration (< 1 mm, 50 g/m^2^). MPDSM (inoculum rate of 100 mL/m^2^) was added to a barley inter-root soil treated with a high concentration of MPs to analyze its effect on inter-root fungi and barley nutrients. Two treatment groups were available. In addition, a control group and a treatment group with only MPDSM added were set up. All treatment groups were cultivated under the same conditions. Each experimental area spanned an area of one square meter, and the experiment lasted for seven months. Inter-root soil samples were collected when plants are mature. Three replicates were made for each treatment group, and the same treatment groups were not placed next to one another.

### 2.3 Recovery and characterization of microplastics

At the end of the experiment, soils from the different treatment groups, as well as from the control group, were collected using the same method. The weights were kept constant, and the recovered MPs were then evaluated by density flotation. Separation was achieved by exploiting the density differences between MPs, soil particles, and a saturated sodium chloride (NaCl) solution (approximately 1.2 g/cm3). A predetermined amount of soil was thoroughly blended with the saturated NaCl solution at a soil-to-solution ratio of 1:5 (w/v). The mixture was homogenized by stirring and subsequently subjected to ultrasonication to ensure complete dispersion. This process allowed the soil particles to settle while the lower-density MPs remained suspended in the solution. The MP-containing suspension was then carefully filtered through a series of organic membrane filters with graded pore sizes for particle size fractionation. The suspension containing MPs was filtered through organic filters of varying pore sizes, allowing for particle size fractionation. An examination of the surface morphology of the sieved PS-MP was conducted using a scanning electron microscope (Sigma300, Zeiss, Germany). With this objective in mind, a thin layer of gold was applied using argon sputtering at 0.3 MPa with a current of 25 mA. The sample was then scrutinized at 10,000 × magnification for surface topography and bacterial growth analysis on MPs.

### 2.4 Determination of nutrients

The nutritional composition of highland barley grains was analyzed using standard methods. The summary of analytical methods is presented in Supplementary Table S1 (Zhang et al., 2021a; Teslová et al., 2010; Huang et al., 2015; Khatri and Chhetri, 2020; Lin et al., 2018; Li et al., 2022b).

### 2.5 Sample collection and DNA extraction

A sample of rhizosphere soil was taken from a barley field in Jianyang, Sichuan, to measure the impact of MPs. Soil samples were collected at three contamination levels (1, 10, and 50 g/m^2^) and two particle sizes (1–5 mm, and < 1 mm). Samples of the large particle size concentrations were named B1, B10, and B50, while the small particle size concentrations were named S1, S10, and S50. To assess the impact of MPDSM addition on inter-root fungi, samples were collected from the MPDSM+MPS treatment group, the control group, and the MPDSM-only treatment group. Naming is as follows: CKA1, only MPDSM treatment soil; BA1, large particle size, high concentration (1–5 mm, 50 g/m^2^), inoculated MPDSM treatment soil; SA1, small particle size, high concentration (< 1 mm, 50 g/m^2^), inoculated MPDSM treatment soil; CK, the control group. The research encompassed three biological replicates per sample, utilizing an ice pack for transportation to the lab for extraction and sequencing of the ITS rDNA. Genomic DNA was extracted from soil samples employing a Soil DNA Kit (Cat. #D5625, Omega Bio-Tek, Norcross, GA, USA). After electrophoresis in a 1% agarose gel, this DNA was diluted to 1 ng/μL in sterilized water.

### 2.6 PCR amplicon

Barcoded printers, named ITS5-1737-F 5′-GGAAGTAAAAGTCGTAACAAGG3′) and ITS2-2043-R 5′-GCTGGTTCATT CATCGATGC3′), contain the ITS1 fragment in the sample collection (Rousk et al., 2010). Fifteen microliters of Phusion^®^ High-Fidelity PCR Master Mix (New England Biolabs) and primers (2 μM each) were mixed with 10 ng of DNA for PCR amplification. Cycling conditions included an initial denaturation step at 98 °C for 1 min, followed by 30 cycles of: denaturation at 98 °C for 10 s, annealing at 50 °C for 30 s, extension at 72 °C for 30 s, then a final extension phase at 72 °C for 5 min. A 2% agarose gel electrophoresis is utilized for PCR product detection. Equal concentrations of the amplicons are purified using a QIAGEN Gel Extraction Kit (QIAGEN, Germany), ensuring purity.

### 2.7 Libraries prepared, sequenced, and processed

Employing the TruSeq^®^ DNA PCR-Free Sample Preparation Kit as per manufacturer guidelines, we produced sequencing libraries incorporating index tags. The library quality was assessed using both the Thermo Scientific Qubit^®^ 2.0 Fluorometer and the Agilent Bioanalyzer 2100 System. The NovaSeq Illumina platform sequenced the library, yielding 250 bp paired-end reads. Utilizing our barcoding technology, we assigned the paired-end sequencing data to individual specimens and truncated them by discarding the barcode and primer information. These paired-end reads were subsequently integrated into raw tags via FLASH software (Magoc and Salzberg, 2011). QIIME 2 quality control protocol was utilized for applying quality filtration on raw tags, meeting pre-determined criteria for pure, superior tags (Caporaso et al., 2010). A tag audit utilizing the referenced Silva Database identified chimera entities, subsequently excised yielding efficient tags for subsequent evaluation (Quast et al., 2013).

### 2.8 OTU cluster and species annotation

Uparse v7.0.1001 was utilized for sequence analysis (Edgar, 2013). OTUs were assigned to any sequence with a similarity of 97% or higher. Sequences representing individual OTUs underwent detailed taxonomic annotation utilizing both the Silva (Quast et al., 2013) and Unite databases (Nilsson et al., 2019). Phylogenetic analysis revealed differences in the dominant species across various samples. MUSCLE v3.8.31 was used for a multiple sequence alignment (Edgar, 2004). Normalization of OTU abundance data was conducted according to the lowest sample count, quantified via sequencing reads. All further analyses were conducted using the normalized output data.

### 2.9 Alpha and beta diversity analyses

The alpha diversity of the rhizosphere fungal communities was assessed using six indices: Observed species, Chao1, Shannon, Simpson, Pielou_e, and Good's coverage, to comprehensively evaluate species richness and evenness within each sample. All alpha diversity indices were calculated based on the rarefied OTU table using QIIME 2. Beta diversity analysis, which examines the compositional differences between microbial communities across samples, was performed based on Bray-Curtis distance matrices. Non-metric multidimensional scaling (NMDS) was conducted using the veganpackage (version 2.15-3) in R to visualize the dissimilarities between sample groups (Caporaso et al., 2010).

### 2.10 Functional predictive modeling and correlation evaluations

FUNGuild was employed for the classification of soil fungal guilds (Nguyen et al., 2016). This procedure began with Principal Component Analysis (PCA) applied through the FactoMineR package and the ggplot2 package within R v2.15.3, reducing the dimensions of the initial variables (Almeida et al., 2018).

### 2.11 Statistical analysis

Results reported via mean ± SD. Data was analyzed by SPSS 19 with ANOVA, including a two-way ANOVA for group comparisons. Pearson correlation assessed linkages between nutrient variables and inter-root fungal counts. Fungal community shifts appraised through NMDS. Statistical significance is set at *P* < 0.05.

## 3 Results

### 3.1 Recycling and characterization of microplastics

**Figure 1:**
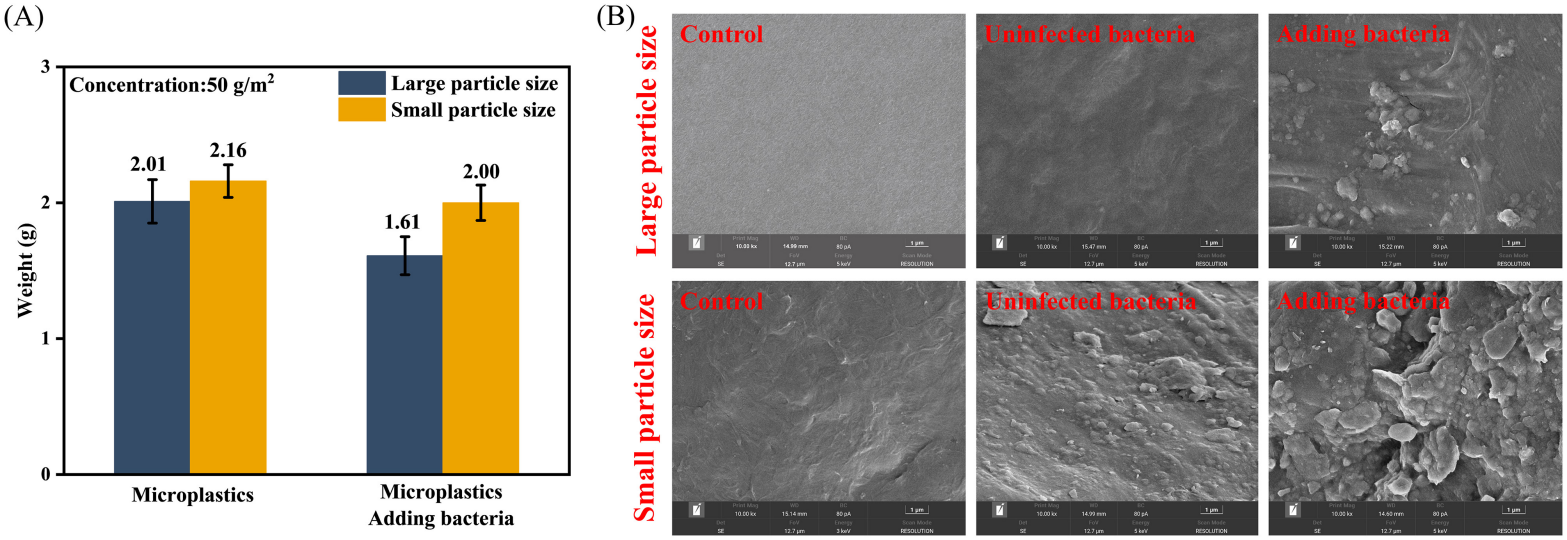
Changes in the weight and morphology of microplastics before and after inoculation. **(A)** Weight loss rate of PS-MPs after inoculation. **(B)** Structural changes in the microplastic surface observed by Scanning Electron Microscope.

At the end of the experiment, MPs were recovered, washed, and dried from the planting soil, and the dry weight was measured. We found that, after adding MPDSM, the weight loss rate of large MP particles was 19.9%, and the weight loss rate of small MP particles was 7.4% (Figure 1A). We performed SEM observation of the recovered MPs (Figure 1B). The surface of the large-particle MPs under unadded MPDSM had a few cracks, and the surface of the small-particle MPs had visible cracks, pores, erosion, and grooves. The surfaces of MPs in the control group appeared smoother. The surface of MPs with MPDSM added had more pronounced grooves, cracks, and pores than the one without MPDSM treatment, indicating the presence of bacterial degradation. The surface roughness of the small MPs was greater than that of the large MPs.

### 3.2 Analysis of the nutritional indicators and mineral element contents of highland barley

The contents of nutrients and mineral elements (total starch, total protein, fat, total sugar, β-glucan, total flavonoids, total polyphenols, vitamin C, vitamin E, potassium, calcium, copper, magnesium, zinc, sodium, and manganese) in highland barley were measured. As shown in Table 1, MP treatment led to significant differences in nutritional indicators, except for starch (*P* < 0.05). In comparison to the control group, the treatment with large particle size MPs significantly elevated the total flavonoid content (*P* < 0.05), with the highest increase of 11.63%, and decreased the contents of total starch, fat, total polyphenols, and vitamin C, with the highest reductions of 2.85%, 14.16%, 12.20%, and 14.41%, respectively. Small particle size MPs treatment remarkably improved the total polyphenol and vitamin E content by 23.17% and 22.21%, respectively (*P* < 0.05), and decreased the contents of fats and vitamin C by up to 19.91% and 8.10%, respectively. In comparison to the control group, the low concentration and small particle size (1 g/m^2^) treatment significantly increased the β-glucan content. Analysis of nutrient element content showed that, in comparison to the control group, large particle size MP significantly reduced levels of Ca, Zn, and Na, while having minimal impact on K, Cu, Mg, and Mn; whereas, small particle size MP significantly increased Zn content, with no observable change in Ca, Mg, or Na (*P* < 0.05).

**Table 1:** Nutrient and mineral content of highland barley grains under microplastic treatment.

**Nutrient**	**Control**	**Large particle size (g/m** ^**2**^ **)**			**Small particle size (g/m** ^**2**^ **)**		
		**1**	**10**	**50**	**1**	**10**	**50**
Starch (%)	43.396 ± 3.068A^(a)^	43.360 ± 2.162A	43.321 ± 0.472A	42.158 ± 0.204A	41.946 ± 1.615^a^	41.017 ± 0.305^a^	40.352 ± 2.100^a^
Crude protein (%)	2.673 ± 0.006B^(b)^	2.685 ± 0.013B	2.917 ± 0.064A	2.944 ± 0.106A	2.933 ± 0.309^ab^	3.048 ± 0.160^ab^	3.263 ± 0.227^a^
Fat (%)	2.260 ± 0.250A^(a)^	2.240 ± 0.020A	2.140 ± 0.290A	1.940 ± 0.340A	1.910 ± 0.100^b^	1.910 ± 0.200^b^	1.810 ± 0.020^b^
Total sugar (%)	25.976 ± 0.094B^(c)^	27.564 ± 0.400B	33.711 ± 0.575A	34.207 ± 0.841A	35.880 ± 0.910^b^	39.211 ± 0.944^a^	28.130 ± 1.302^c^
β-Glucan (%)	5.179 ± 0.120B^(b)^	5.258 ± 0.119B	5.286 ± 0.040B	5.591 ± 0.050A	5.464 ± 0.120^a^	5.386 ± 0.022^ab^	5.208 ± 0.124^b^
Total flavonoids (%)	0.215 ± 0.002D^(a)^	0.230 ± 0.001C	0.234 ± 0.001B	0.240 ± 0.001A	0.219 ± 0.008^a^	0.243 ± 0.005^b^	0.253 ± 0.008^b^
Total polyphenols (umol/g)	9.950 ± 0.093A^(c)^	9.864 ± 0.150A	9.839 ± 0.021A	8.736 ± 0.064B	10.112 ± 0.098^b^	10.223 ± 0.037^b^	12.255 ± 0.020^a^
Vitamin C (%)	0.111 ± 0.003A^(a)^	0.106 ± 0.001B	0.104 ± 0.003B	0.095 ± 0.001C	0.110 ± 0.001^a^	0.104 ± 0.001^b^	0.102 ± 0.002^b^
Vitamin E (mg/100g)	1.342 ± 0.026B^(a)^	1.370 ± 0.043B	1.394 ± 0.023B	1.520 ± 0.017A	1.632 ± 0.070^b^	1.639 ± 0.029^b^	1.640 ± 0.034^b^
K	4.670 ± 0.126A^(ab)^	4.489 ± 0.305A	4.801 ± 0.0586A	4.691 ± 0.373A	5.007 ± 0.274^a^	4.117 ± 0.295^b^	4.999 ± 0.422^a^
Ca	0.556 ± 0.061A^(a)^	0.422 ± 0.018B	0.464 ± 0.026B	0.468 ± 0.037B	0.508 ± 0.036^a^	0.487 ± 0.181^a^	0.587 ± 0.085^a^
Cu	0.209 ± 0.008A^(a)^	0.175 ± 0.053A	0.179 ± 0.018A	0.165 ± 0.046A	0.156 ± 0.022^b^	0.113 ± 0.013^c^	0.179 ± 0.019^ab^
Mg	0.825 ± 0.027A^(a)^	0.791 ± 0.010A	0.793 ± 0.055A	0.803 ± 0.050A	0.788 ± 0.031^a^	0.753 ± 0.060^a^	0.796 ± 0.044^a^
Mn	6.585 ± 0.069A^(b)^	6.523 ± 0.325A	6.778 ± 0.219A	6.797 ± 0.588A	7.032 ± 0.215^ab^	6.597 ± 0.373^b^	7.326 ± 0.281^a^
Zn	0.943 ± 0.016A^(a)^	0.751 ± 0.046B	0.749 ± 0.043B	0.730 ± 0.110B	0.663 ± 0.101^b^	0.430 ± 0.025^c^	0.434 ± 0.031^c^
Na	0.189 ± 0.053A^(a)^	0.114 ± 0.022B	0.109 ± 0.001B	0.109 ± 0.054B	0.091 ± 0.036^a^	0.099 ± 0.423^a^	0.138 ± 0.061^a^

Each sample contains 3 biological replicates, and the values are expressed as mean ± standard deviation. Uppercase letters represent large particle-size treatment groups, while lowercase letters represent small particle-size treatments. Different letters in different lines indicate significant differences between different treatments (P < 0.05).

Table 2 shows the nutrient and mineral content of highland barley inoculated with the MPDSM. In comparison to the control group, the inoculation with the only MPDSM treatment significantly increased the content of total proteins, total sugars, total flavonoids, and total polyphenols, increasing by 21.44%, 45.79%, 18.14%, and 28.6%, respectively; the large-particle-size MP inoculation with the MPDSM treatment significantly increased the content of total proteins, total sugars, β-glucans, total flavonoids, and total polyphenols by 25.14%, 27.81%, 4.94%, 12.09%, and 22.17%, respectively; and significantly reduced the total starch content by 14.75% (*P* < 0.05). In comparison to the MP treatment group utilizing larger particles, inoculation with the MPDSM significantly increased the content of fat, total polyphenols, and vitamin C by 32.01%, 39.15%, and 10.53%, respectively. The contents of starch, β-glucans, and vitamin E were reduced by 12.24%, 2.8%, and 12.70%, respectively (*P* < 0.05). In comparison to the control group, the inoculation with the MPDSM of small particle-size MPs significantly increased the content of total sugars, β-glucans, total flavonoids, total polyphenols, vitamin C, and vitamin E by 55.15%, 4.56%, 6.98%, 15.70%, 7.21%, and 6.86%, respectively, reducing the total starch content by 21.11% (*P* < 0.05). In comparison to the MP treatment group utilizing small particles, inoculation with the MPDSM significantly increased the content of fat, total sugars, β-glucans, and vitamin C by 30.39%, 43.55%, 3.97%, and 16.67%, respectively. The contents of total protein, total flavonoids, total polyphenols, and vitamin E were reduced by 16.92%, 9.09%, 6.06%, and 12.61%, respectively (*P* < 0.05). Analysis of nutrient element content showed that the inoculation with the MPDSM treatment significantly diminished levels of Ca and Zn, leaving levels of K, Cu, Mg, Mn, and Na unaffected in comparison to the control (*P* < 0.05). The zinc content in the large-size MP inoculation with the MPDSM treatment group was significantly elevated compared to the MP treatment group, whereas there was no significant effect in the small-size MP inoculation with the MPDSM treatment group (*P* < 0.05).

**Table 2:** Nutrient and mineral content of highland barley grains under inoculation microplastic-degrading synthetic microbiota treatment.

**Nutrient**	**Control**	**Inoculation only**	**Large particle size (50g/m** ^**2**^ **)**		**Small particle size (50g/m** ^**2**^ **)**	
			**Microplastics**	**Microplastics and Inoculation**	**Microplastics**	**Microplastics and Inoculation**
Starch (%)	43.396 ± 3.068A^(a)^	44.550 ± 3.634A^(a)^	42.158 ± 0.204A	36.997 ± 3.985B	40.352 ± 2.101^b^	34.234 ± 3.918^b^
Crude protein (%)	2.673 ± 0.006B^(b)^	3.246 ± 0.336A^(a)^	2.943 ± 0.106AB	3.345 ± 0.371A	3.263 ± 0.226^a^	2.711 ± 0.384^b^
Fat (%)	2.260 ± 0.250AB^(a)^	1.960 ± 0.190B^(ab)^	1.940 ± 0.340B	2.561 ± 0.190A	1.810 ± 0.021^b^	2.360 ± 0.311^a^
Total sugar (%)	25.976 ± 0.582 C^(d)^	37.871 ± 0.279 A^(b)^	34.207 ± 0.841B	33.200 ± 0.841B	28.130 ± 1.302^c^	40.380 ± 1.824^a^
β-Glucan (%)	5.179 ± 0.120C^(b)^	5.283 ± 0.051C^(ab)^	5.591 ± 0.0480A	5.435 ± 0.068B	5.208 ± 0.124^b^	5.415 ± 0.0146^a^
Total flavonoids (%)	0.215 ± 0.002C^(c)^	0.254 ± 0.007A^(a)^	0.240 ± 0.001B	0.241 ± 0.004B	0.253 ± 0.008^a^	0.230 ± 0.004^b^
Total polyphenols (umol/g)	9.950 ± 0.093C^(c)^	12.797 ± 0.353A^(a)^	8.736 ± 0.064D	12.156 ± 0.304B	12.255 ± 0.02^a^	11.512 ± 0.485^b^
Vitamin C (%)	0.111 ± 0.003A^(b)^	0.105 ± 0.005A^(bc)^	0.095 ± 0.001B	0.105 ± 0.003A	0.102 ± 0.00^c^	0.119 ± 0.002^a^
Vitamin E (mg/100 g)	1.342 ± 0.026B^(c)^	1.327 ± 0.001B^(c)^	1.520 ± 0.017A	1.327 ± 0.001B	1.641 ± 0.034^a^	1.434 ± 0.026^b^
K	4.670 ± 0.126A^(b)^	4.605 ± 0.362A^(b)^	4.691 ± 0.373A	4.933 ± 0.106A	4.999 ± 0.422^b^	4.477 ± 0.206^b^
Ca	0.556 ± 0.061A^(a)^	0.403 ± 0.093B^(b)^	0.468 ± 0.037AB	0.580 ± 0.042A	0.587 ± 0.085^a^	0.623 ± 0.049^a^
Cu	0.209 ± 0.008A^(a)^	0.181 ± 0.050A^(a)^	0.165 ± 0.046A	0.207 ± 0.006A	0.179 ± 0.019^a^	0.156 ± 0.043^a^
Mg	0.825 ± 0.027A^(a)^	0.809 ± 0.034A^(a)^	0.803 ± 0.050A	0.806 ± 0.033A	0.796 ± 0.044^a^	0.770 ± 0.022^a^
Mn	6.585 ± 0.069A^(a)^	6.519 ± 0.665A^(a)^	6.797 ± 0.588A	7.336 ± 0.724A	7.326 ± 0.281^a^	7.075 ± 0.398^a^
Zn	0.943 ± 0.016A^(a)^	0.673 ± 0.121B^(a)^	0.730 ± 0.110B	1.062 ± 0.052A	0.434 ± 0.031^a^	0.781 ± 0.164^a^
Na	0.189 ± 0.053A^(a)^	0.164 ± 0.015AB^(a)^	0.109 ± 0.054B	0.120 ± 0.154AB	0.138 ± 0.061^a^	0.163 ± 0.031^a^

Each sample contains 3 biological replicates, and the values are expressed as mean ± standard deviation. Uppercase letters represent large particle-size treatment groups, while lowercase letters represent small particle-size treatments. Different letters in different lines indicate significant differences between different treatments (P < 0.05).

### 3.3 Rhizosphere soil fungal diversity

#### 3.3.1 Sequencing data analysis

Using high-throughput sequencing, we analyzed changes in inter-root fungal biodiversity and community structure at three concentration levels and two different particle sizes of MPs, as well as under treatment with inoculated with the MPDSM. The OTU dilution curves are shown in Supplementary Figure S1. Subject to an increase in sequencing read volume, observed species rise until they progress beyond 46,924 reads, where the rarefaction curve levels off as a sufficient representation of the soil fungal community's structure is ascertained. Following deduplication and quality assessment, a total of 52,789 clean reads remained per sample, suitable for subsequent analyses. Establishing a 97% similarity threshold, these reads were classified into OTUs; the range varied between 241 and 1,072 per sample, with an overall average of 521 OTUs across all samples.

#### 3.3.2 Sample categorization and abundance

**Figure 2:**
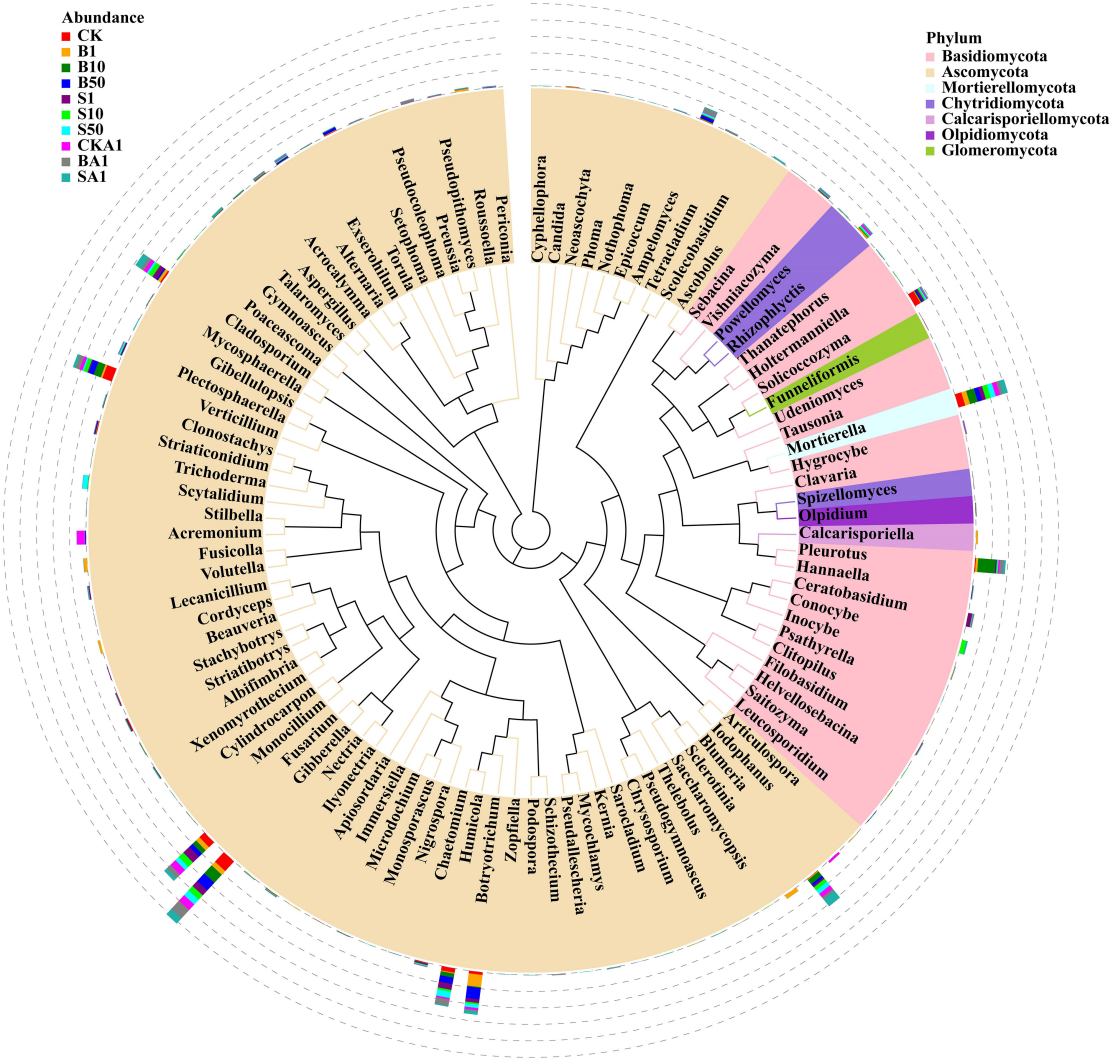
Phylogenetic relationship and abundance of the 100 most abundant fungal genera in different samples. CK, MPs-free, uninoculated microplastic-degrading synthetic microbiota (MPDSM) soil; B1, large particle size, low concentration soil (1–5 mm, 1 g/m^2^); B10, large particle size, medium concentration soil (1–5 mm, 10 g/m^2^); B50, large particle size, high concentration soil (1–5 mm, 50 g/m^2^); S1, small particle size, low concentration soil (<1 mm, 1 g/m^2^); S10, small particle size, medium concentration soil (<1 mm, 10 g/m^2^); S50, small particle size, high concentration soil (<1 mm, 50 g/m^2^); CKA1, only inoculated MPDSM soil; BA1, large particle size, high concentration (1–5 mm, 50 g/m^2^), inoculated MPDSM soil; SA1, small particle size, high concentration (<1 mm, 50 g/m^2^), inoculated MPDSM soil.

Categorized by fungi, the study identified a total of 12 phyla, 42 classes, 98 orders, 229 families, and 497 genera from all analyzed samples. Ascomycota notably dominated all sample compositions, with Basidiomycota as a secondary contributor. Sordariomycetes dominated at the class level, closely followed by Agaricomycetes. Otherwise, the predominant fungi were Agaricales, followed by Hypocreales. At the family level, the Pleurotaceae topped the charts, with the Chaetomiaceae close behind. *Pleurotus* dominated in sample abundance at the genus level, followed by *Botryotrichum*. Figure 2 illustrates the classification of the predominant fungi in all trials.

#### 3.3.3 Alpha diversity indices

**Figure 3:**
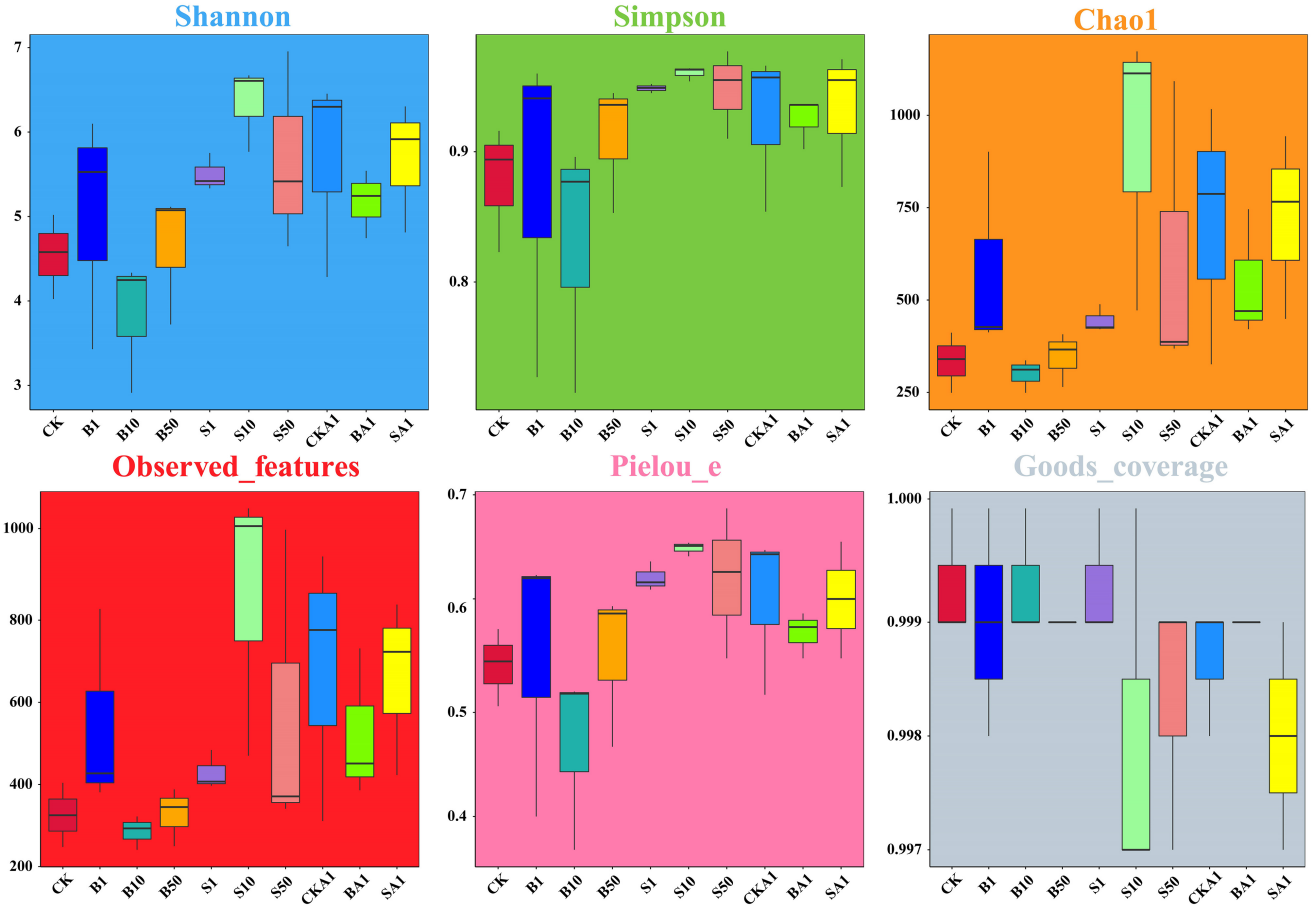
Box plot of the fungal α-diversity index in different samples. CK, MPs-free, uninoculated microplastic-degrading synthetic microbiota (MPDSM) soil; B1, large particle size, low concentration soil (1–5 mm, 1 g/m^2^); B10, large particle size, medium concentration soil (1–5 mm, 10 g/m^2^); B50, large particle size, high concentration soil (1–5 mm, 50 g/m^2^); S1, small particle size, low concentration soil (< 1 mm, 1 g/m^2^); S10, small particle size, medium concentration soil (< 1 mm, 10 g/m^2^); S50, small particle size, high concentration soil (< 1 mm, 50 g/m^2^); CKA1, only inoculated MPDSM soil; BA1, large particle size, high concentration (1–5 mm, 50 g/m^2^), inoculated MPDSM soil; SA1, small particle size, high concentration (< 1 mm, 50 g/m^2^), inoculated MPDSM soil.

The species diversity was analyzed using six indices, including the Shannon, Simpson, Chao1, Observed-Features, Pielou_e, and Good_Coverage indices (Figure 3). Our community structure biodiversity (Shannon, Simpson, observed species, and Chao1 statistics) analysis indicated that the small-grain, intermediate concentration S10 samples exhibited the highest biodiversity and richness rates. Conversely, the large-grain, intermediate-concentration B10 samples displayed the poorest biodiversity and richness scores. Except for the B10 sample, the Simpson, Shannon, observed species, and Chao1 indices of the other samples surpass the CK group's values. Nevertheless, only the S10 sample presents substantial disparity from the CK group on diversity and richness indices (*P* < 0.05). Analysis of inoculation with the MPDSM treatment indicated that, while BA1 and SA1 exhibited diminished diversities relative to CKA1, SA1 fostered a richer biodiversity than BA1. The sample homogenization indicated that S10 exhibited superior homogeneity, while B10 displayed the least. The homogeneity analysis of the inoculation with the MPDSM treatment showed that the BA1 and SA1 samples were less homogeneous than the CKA1 group and that BA1 was less homogeneous than SA1. In terms of sequence depth, all samples presented substantial sequencing depth (averaged over a Good Coverage Index of >0.997).

#### 3.3.4 Fungal community taxonomy

**Figure 4:**
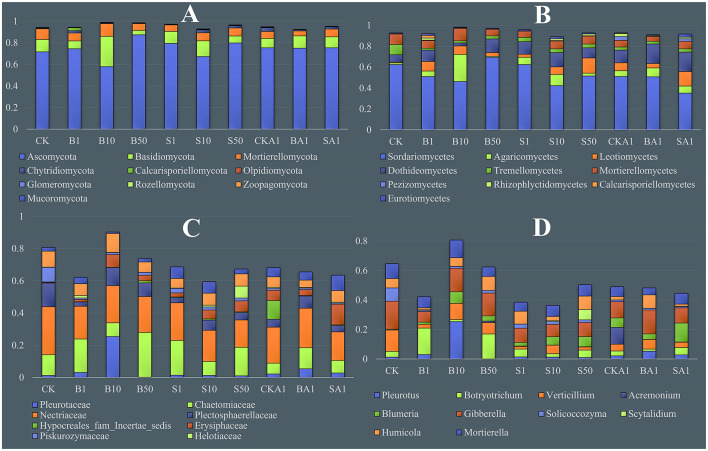
Relative abundance of taxa (top 10) at the Phylum **(A)**, Class **(B)**, Family **(C)**, and Genus **(D)** levels. CK, MPs-free, uninoculated microplastic-degrading synthetic microbiota (MPDSM) soil; B1, large particle size, low concentration soil (1–5 mm, 1 g/m^2^); B10, large particle size, medium concentration soil (1–5 mm, 10 g/m^2^); B50, large particle size, high concentration soil (1–5 mm, 50 g/m^2^); S1, small particle size, low concentration soil (< 1 mm, 1 g/m^2^); S10, small particle size, medium concentration soil (< 1 mm, 10 g/m^2^); S50, small particle size, high concentration soil (< 1 mm, 50 g/m^2^); CKA1, only inoculated MPDSM soil; BA1, large particle size, high concentration (1–5 mm, 50 g/m^2^), inoculated MPDSM soil; SA1, small particle size, high concentration (< 1 mm, 50 g/m^2^), inoculated MPDSM soil.

The experiment evaluated a shift in the occurrence of the top ten fungal phyla among all sample regions (Figure 4A). The Ascomycota phylum was dominant in all samples, representing approximately 74.5% of the fungi surveyed. Compared with CK (71.7% on average), the abundance of Ascomycota was increased in the B1, B50, S1, S50, CKA1, BA1, and SA1 samples, with the highest abundance in the B50 sample (87.5% on average). Ascomycota abundance was reduced in B10 and S10 samples, with the lowest abundance of Ascomycota in B10 samples (average 58.1%). No distinct change in Ascomycota abundance was observed between the CKA1 group and the BA1/SA1 samples (*P* < 0.05). In all soil samples, the Basidiomycota phylum was the predominant one, at a proportion of 11.3%. This was followed by Basidiomycota (average 7.4%) and Chytridiomycota (average 1.5%). The abundance of Basidiomycota increased in B10 samples compared to the CK group, while the abundance of Ascomycota decreased. Inoculation with the MPDSM treatment samples showed an increase in Ascomycota and Chytridiomycota abundance, and a decrease in Basidiomycota and Mortierellomycota compared to the CK.

Overall, analysis of the rhizosphere soil ascertained the presence of 42 diverse fungal taxa at the class level (Figure 4B). The most abundant sample was Sordariomycetes (average 52.3%), followed by Dothideomycetes (average 11.9%), Agaricomycetes (average 7.5%), Mortierellomycetes (average 7.4%), and Leotiomycetes (average 7.2%). There was an increase in members of the Sordariomycetes in B50 and S1 samples compared to CK, however, an evident decrease occurred in CKA1, BA1, and SA1 (*P* < 0.05). In contrast to CKA1, the levels of Sordariomycetes declined in BA1 and SA1, while those of Agaricomycetes and Dothideomycetes increased. Substantially, the Leotiomycetes count increased universally across all treatments, whereas the Tremellomycetes decreased compared to the CK group.

The four families that were most abundant across all samples were Nectriaceae (22.0%), Chaetomiaceae (14.5%), Plectosphaerellaceae (6.8%), and Pleurotaceae (4.5%) (Figure 4C). Compared with the CK, the MP, and inoculation with the MPDSM treatment significantly reduced the abundance of Nectriaceae and Plectosphaerellaceae in the rhizosphere soil (*P* < 0.05), with the lowest content of Nectriaceae in the S50 sample (16.9%) and the lowest content of Plectosphaerellaceae in the B1 sample (2.7%). In contrast with CK1, both Pleurotaceae and Chaetomiaceae species accumulate significantly more in BA1 and marginally higher in SA1 (*P* < 0.05).

Among genera, *Gibberella* dominated all samples, followed by *Botryotrichum, Verticillium*, and *Blumeria* (Figure 4D). In comparison to the control group, the MPs and inoculation with the MPDSM treatments significantly increased the abundance of *Blumeria* in the rhizosphere soil, with the highest abundance in the B1 sample; the contents of *Gibberella* and *Verticillium* were decreased (*P* < 0.05). Compared with controls, those inoculated with the MPDSM treatments significantly amplified populations of *Blumeria* and *Pleurotus*, substantially reducing those of *Botryotrichum, Verticillium*, and *Gibberella* (*P* < 0.05). Among inoculated samples with the MPDSM treatment, *Gibberella* was the highest in the BA1 sample, *Botryotrichum* was the highest in the SA1 sample, and *Blumeria* was the highest in the CKA1 sample.

#### 3.3.5 Fungal community structure differentiation

Exploring sample specificities and shared OTUs in this research (Supplementary Figure S2). Fungal root communities of B1, B10, and B50 exposed to varying large particle concentrations displayed 1,115, 319, and 372 unique OTUs, respectively, relative to the CK, while their corresponding inter-root communities enriched with small grains portrayed 577, 1,730, and 1,127 unique OTUs, respectively (Supplementary Figure S2). A coexistence of 204 core OTUs was witnessed for B1, B10, and B50; whereas 272 core OTUs were observed within the samples S1, S10, and S50. Compared to CKA1, the rhizosphere fungi of B50 and BA1 presented 364 and 774 unique OTUs, respectively; the fungi of S50 and SA1 displayed 862 and 1,014 unique OTUs, respectively. Notably, both B50 and BA1 shared 309 core OTUs, while the S50 and SA1 harbored 542 core OTUs. In total, the rhizosphere fungal community samples treated with MPs contained 150 core OTUs, and each sample contained 173 to 1,151 unique OTUs. The inoculated rhizosphere samples with the MPDSM treatment contained 164 core OTUs, and each sample contained 217 to 810 unique OTUs (Supplementary Figure S2).

**Figure 5:**
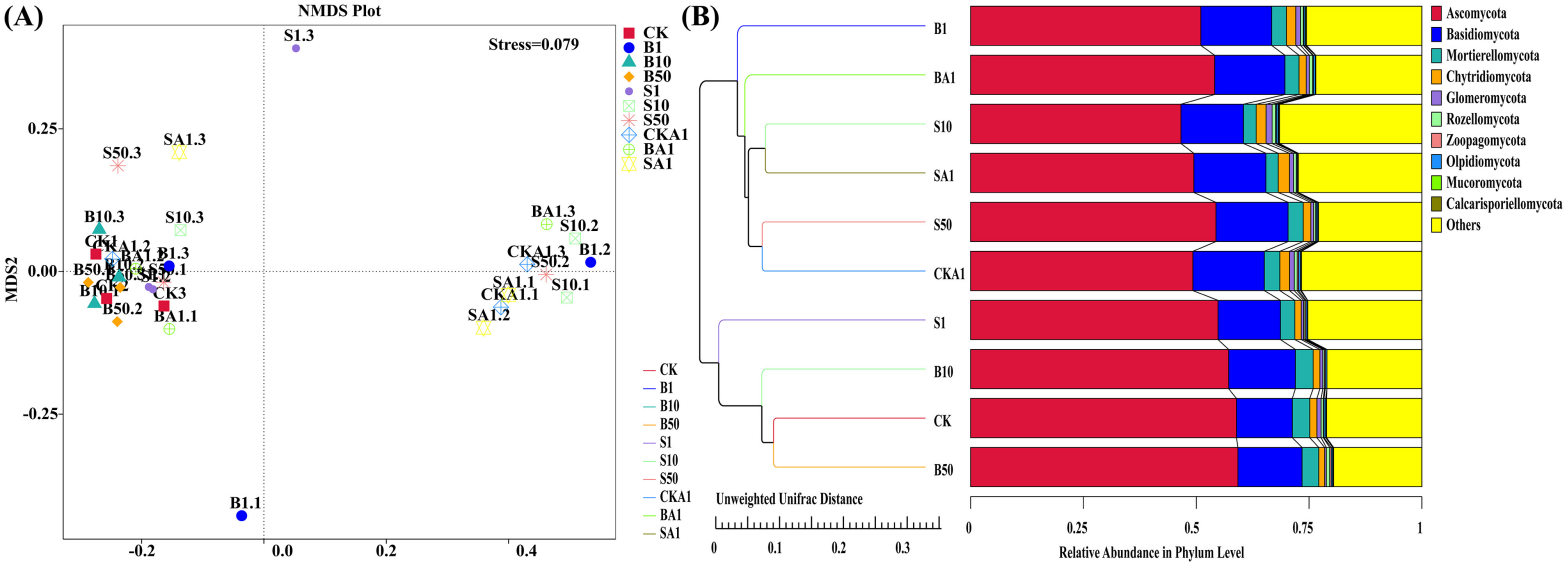
Analysis of community β-diversity between different samples based on NMDS analysis **(A)** and weighted UniFrac distance **(B)**. CK, MPs-free, uninoculated microplastic-degrading synthetic microbiota (MPDSM) soil; B1, large particle size, low concentration soil (1–5 mm, 1 g/m^2^); B10, large particle size, medium concentration soil (1–5 mm, 10 g/m^2^); B50, large particle size, high concentration soil (1–5 mm, 50 g/m^2^); S1, small particle size, low concentration soil (< 1 mm, 1 g/m^2^); S10, small particle size, medium concentration soil (< 1 mm, 10 g/m^2^); S50, small particle size, high concentration soil (< 1 mm, 50 g/m^2^); CKA1, only inoculated MPDSM soil; BA1, large particle size, high concentration (1–5 mm, 50 g/m^2^), inoculated MPDSM soil; SA1, small particle size, high concentration (< 1 mm, 50 g/m^2^), inoculated MPDSM soil.

A comparative analysis of fungal respective communities in various samples was conducted using NMDS (Figure 5). Community similarity was higher in the CK samples and the samples treated with large grain sizes, B10 and B50, compared to the other samples. Inoculating with the MPDSM treatment altered the structure of the rhizosphere fungal community. The similarity between Sample BA1 and B1 was higher for samples treated with MPDSM and a large particle size; the similarity between Samples SA1 and S10 was higher for samples treated with MPDSM and a small particle size. NMDS analysis showed that the MP-treated B1 sample had the greatest effect on rhizobia. In contrast, B50 had the least effect.

#### 3.3.6 Fungal community functional prediction

Our prediction of the fungal community's function in soil samples was based on FUNGuild. The function of fungi falls into 64 categories, where saprotrophs bear the most responsibility (35.4%, on average), followed by pathotrophs (26.6%, on average), unassigned (16.9%, on average), pathotrophs-saprotrophs (9.2%, on average), and pathogen-saprotroph-symbiotrophs (1.4%, on average). The taxa with the greatest variation in fungal species were further assessed using PCA (Supplementary Figure S3). The results indicated that the inter-root fungal functions were differentiated to some extent compared to those of the CK samples. Inoculation with the MPDSM treatments resulted in a functional differentiation of the inter-root fungal flora compared to the MP treatment group.

#### 3.3.7 Guild variations

**Figure 6:**
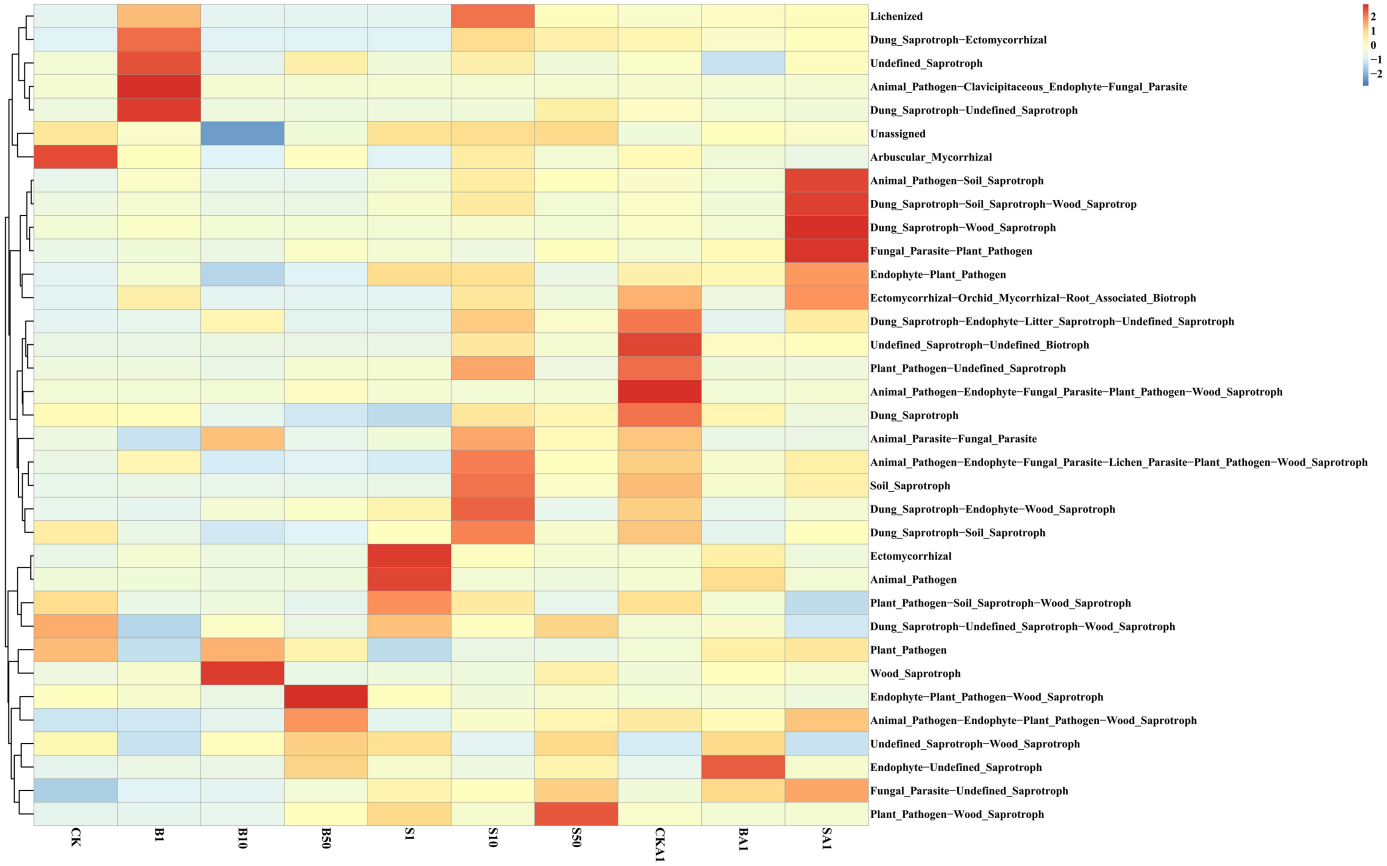
Enrichment heatmap of the functional model between different samples. The closer the color is to red, the higher the abundance; the closer it is to blue, the lower the abundance. CK, MPs-free, uninoculated microplastic-degrading synthetic microbiota (MPDSM) soil; B1, large particle size, low concentration soil (1–5 mm, 1 g/m^2^); B10, large particle size, medium concentration soil (1–5 mm, 10 g/m^2^); B50, large particle size, high concentration soil (1–5 mm, 50 g/m^2^); S1, small particle size, low concentration soil (< 1 mm, 1 g/m^2^); S10, small particle size, medium concentration soil (< 1 mm, 10 g/m^2^); S50, small particle size, high concentration soil (< 1 mm, 50 g/m^2^); CKA1, only inoculated MPDSM soil; BA1, large particle size, high concentration (1–5 mm, 50 g/m^2^), inoculated MPDSM soil; SA1, small particle size, high concentration (< 1 mm, 50 g/m^2^), inoculated MPDSM soil.

**Figure 7:**
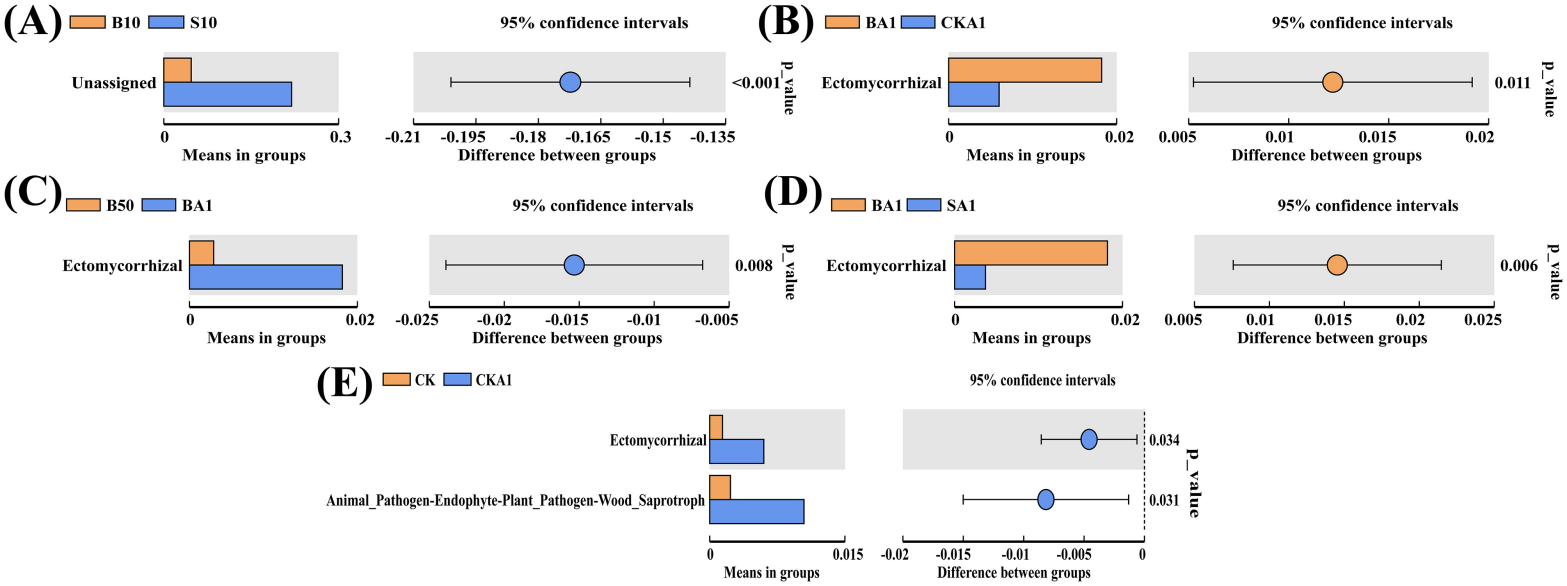
Functional models that are significantly enriched among different samples (*P* < 0.05). **(A)** Comparison between B10 and S10. **(B)** Comparison between BA1 and CKA1. **(C)** Comparison between B50 and BA1. **(D)** Comparison between BA1 and SA1. **(E)** Comparison between CK and CKA1. CK, MPs-free, uninoculated microplastic-degrading synthetic microbiota (MPDSM) soil; B1, large particle size, low concentration soil (1–5 mm, 1 g/m^2^); B10, large particle size, medium concentration soil (1–5 mm, 10 g/m^2^); B50, large particle size, high concentration soil (1–5 mm, 50 g/m^2^); S1, small particle size, low concentration soil (< 1 mm, 1 g/m^2^); S10, small particle size, medium concentration soil (< 1 mm, 10 g/m^2^); S50, small particle size, high concentration soil (< 1 mm, 50 g/m^2^); CKA1, only inoculated MPDSM soil; BA1, large particle size, high concentration (1–5 mm, 50 g/m^2^), inoculated MPDSM soil; SA1, small particle size, high concentration (< 1 mm, 50 g/m^2^), inoculated MPDSM soil.

The function of inter-root fungal communities in soils treated with MPs and inoculated with the MPDSM treatment diverged compared to the CK (Figure 6). Study findings revealed an enhancement in functions for Fungal_Parasite-Undefined_Saprotroph, Animal_Pathogen-Endophyte-Plant_Pathogen-Wood_Saprotroph, and Ectomycorrhizal, while a reduction occurred in Dung_Saprotroph- Undefined_Saprotroph-Wood_Saprotroph and Arbuscular_Mycorrhizal functions. The enrichment of Fungal_Parasite-Undefined_Saprotrophprofiles suggests a potential shift toward a more parasitic and decomposer-oriented fungal community under MP stress, which might reflect an adaptive response to the altered soil environment and could impact plant health and organic matter turnover. In the MP treatment's large particle size group, 1′s Dung_Saprotroph-Ectomycorrhizal and Undefined_Saprotroph surpassed all others, alongside B0′s Animal_Pathogen-Clavicipitaceous_Endophyte-Fungal_Parasite. Endophyte-Plant_Pathogen-Wood_Saprotroph's role in the B50 sample markedly outperformed its counterparts (*P* < 0.05). For the small particle size MP treatment group, the functions of Ectomycorrhizal and Animal_Pathogen in S1 samples, the function of Dung_Saprotroph-Endophyte-Wood_Saprotroph in S10 samples, and the function of Plant_Pathogen-Wood_Saprotroph in S50 samples were higher than those in the other treatment groups. The functions of Undefined_Saprotroph-Undefined_Biotroph in CKA1 samples, and Fungal_Parasite-Plant_Pathogen in SA1 samples, were significantly higher in the group inoculated with the MPDSM treatment, compared to the other groups (*P* < 0.05). In addition, the functions of Animal_Pathogen-Endophyte-Fungal_Parasite-Plant_Pathogen-Wood_Saprotroph in CKA1 samples, Endophyte-Undefined_Saprotroph in BA1 samples, and Animal_Pathogen-Soil_Saprotroph, Dung_Saprotroph-Soil_Saprotroph-Wood_Saprotroph and Dung_Saprotroph in SA1 samples were higher than those of the other treatments. We also found that, compared with B10 samples, the unassigned function was significantly increased in S10 samples (*P* < 0.05) (Figure 7A); when compared with BA1, the ectomycorrhizal functionality notably diminished in CK1 (*P* < 0.05) (Figure 7B). In comparison to B50 samples, the ectomycorrhizal function was significantly elevated in the BA1 samples (*P* < 0.05) (Figure 7C). Compared with BA1 samples, the ectomycorrhizal function was significantly reduced in SA1 samples (*P* < 0.05) (Figure 7D). Significantly lower ectomycorrhizal activity observed in CKA1 compared to CK, while significant elevation of animal pathogen-endophyte–plant pathogen-wood saprotroph activities (*P* < 0.05) (Figure 7E).

### 3.4 Analyzing Pearson correlation

**Figure 8:**
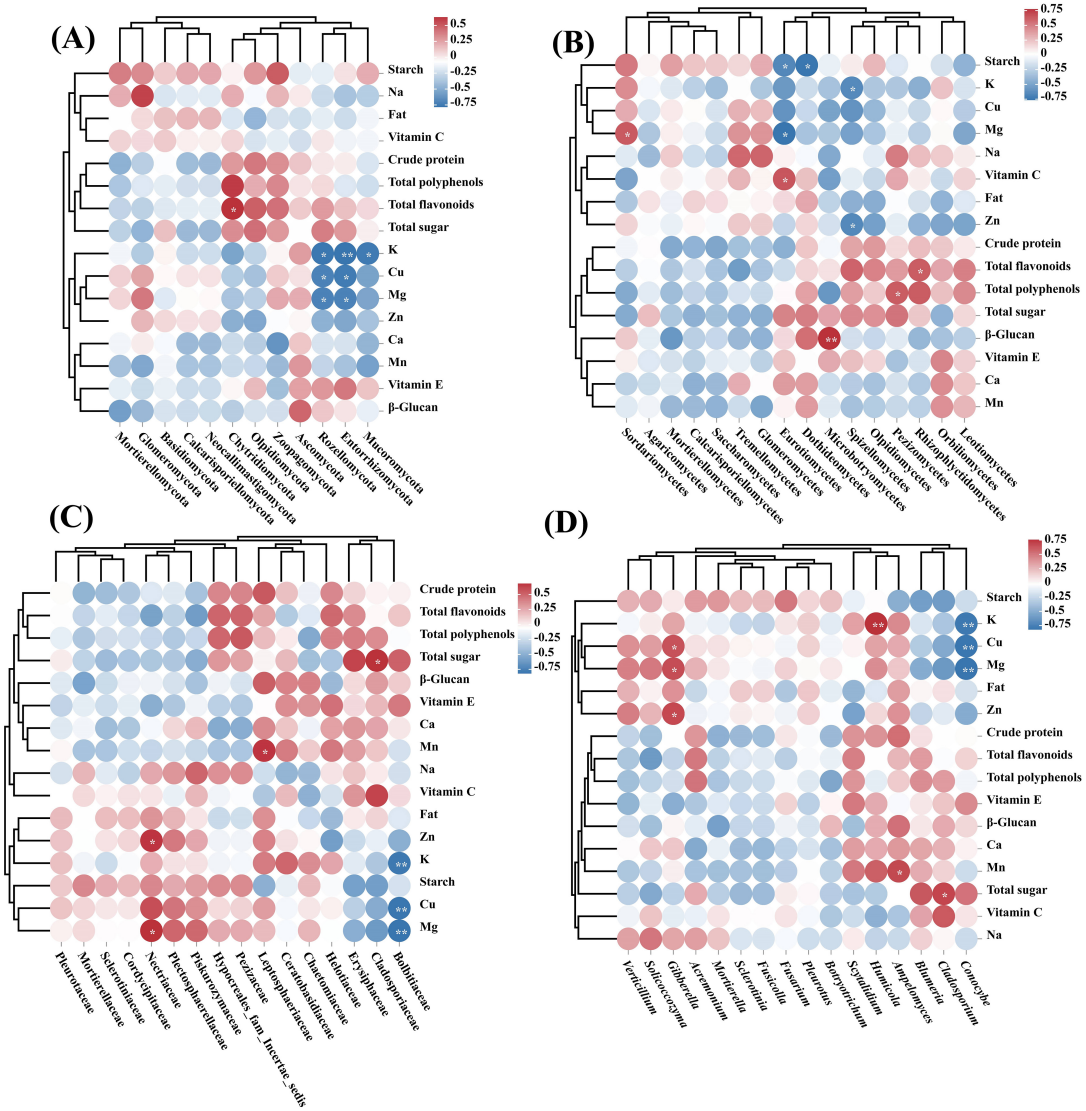
Heatmap of the dynamic correlation between the nutrient indicators of highland barley grain and the fungal biodiversity of the rhizosphere soil under different treatment groups (*P* < 0.05). **(A)** Correlation between trophic indicators and fungal biodiversity at the phylum level. **(B)** Correlation between trophic indicators and fungal biodiversity at the class level. **(C)** Correlation between trophic indicators and fungal biodiversity at the family level. **(D)** Correlation between trophic indicators and fungal biodiversity at the genus level. The closer the color is to red, the higher the positive correlation; the closer it is to blue, the higher the negative correlation. Asterisks in circles represent significant correlations.

Pearson correlation analysis revealed significant correlations between nutrient indexes and inter-root soil fungal diversity in highland barley after treatment with different particle sizes and concentrations of MPs and inoculation with MPDSM (Figure 8). Only the Chytridiomycota abundance showed a significant positive correlation with the total flavonoid content at the phylum level (Figure 8A). In contrast, the abundances of Rozellomycota and Entorrhizomycota had a significant negative relationship with the contents of K, Cu, and Mg; the abundance of Mucoromycota was significantly correlated with the content of K; and there was a significant negative correlation (*P* < 0.05). At the class level (Figure 8B), Saccharomycetes and Mg, Eurotiomycetes and Vitamin C, Microbotryomycetes and β-glucan, Pezizomycetes and Total polyphenols, and Rhizophlyctidomycetes were significantly positively correlated with the Total flavonoid content. The abundance of Eurotiomycetes and Mg and Starch, Dothideomycetes and Starch, and Spizellomycetes were negatively correlated with the contents of K and Zn (*P* < 0.05). Family-level results revealed significant positive associations between Nectriaceae abundance and levels of Zn and Mg, Leptosphaeriaceae abundance and Mn concentration, and Cladosporiaceae abundance and total sugar content. Conversely, the density of Bolbitiaceae negatively correlated significantly with the contents of K, Cu, and Mg (*P* < 0.05) (Figure 8C). At the genus level (Figure 8D), there were significant positive correlations between *Gibberella* and Cu, Mg, and Zn; *Humicola* and K; *Ampelomyces* and Mn; and the abundance of *Cladosporium* and the total sugar content, while only *Conocybe* had an association with K, Cu, and Mg contents with a significant negative correlation (*P* < 0.05).

## 4 Discussion

### 4.1 Degradation effects of microplastics

Artificial synthetic flora could degrade PS-MPs (Xiang et al., 2023c). In this study, it was found that the degradation rate of large-particle MP was higher than that of small-particle MP, which may be due to the larger contact area of large-particle MP, which can attach more bacteria, as well as the influence of the environment that leads to the degradation of large-particle MPs. In contrast, small-particle MPs exhibited more surface erosion under SEM. This apparent paradox can be explained by the higher environmental mobility and bioavailability of small MPs; their smaller size allows them to be more readily dispersed and accessed by microbes for surface degradation, but their higher dispersion might also reduce the local enzyme concentration and sustained contact needed for bulk mass loss, leading to a lower overall mass degradation rate compared to larger particles that, once colonized, can be degraded more efficiently *in situ* (Iqbal et al., 2025). Our previous study found that a mixture of three bacterial strains degraded 17.0% of small-sized PS-MPs within 60 days under laboratory conditions (Xiang et al., 2023c). In contrast, the degradation rate of small-sized PS-MPs in the present experiment was only 7.4% in a large field over up to 7 months, which was much lower than the degradation efficiency under laboratory conditions. This may be related to the antagonism between the inocula and the native bacteria, resulting in a low survival rate of the inocula and thus a reduced degradation efficiency. SEM showed that significant changes appeared on the surface of MPs, which was consistent with our previous study (Xiang et al., 2023c). More pronounced indentations and grooves were observed on the surface of the smaller-sized MPs, due to their higher availability (García-Depraect et al., 2022). Consequently, we affirm that MP dimensions drastically influence their potential for degradation within the soil. More importantly, from laboratory studies to the practical application of microplastic-degrading synthetic microbiota, further studies are needed.

### 4.2 Nutritional indicators and mineral content of highland barley

MP treatment led to significant differences in nutritional indicators, except for starch (*P* < 0.05). Studies have shown that flavonoids and polyphenols are important secondary metabolites in plants, and the increase in their levels plays a crucial role in aiding plants to tolerate, resist, and defend against abiotic stresses (Sharma et al., 2019; Shah and Smith, 2020). In this study, MPs with large particle sizes significantly increased the content of total flavonoids, and MPs with small particle sizes significantly increased the content of total polyphenols (*P* < 0.05), confirming that, after plants are subjected to abiotic stress, different metabolites in plants vary greatly. Starch is a main component in plants, and its content decreases under abiotic stress (Chang and Lv, 2016). The degradation of plant starch into sugars and other derived metabolites supports plant growth under stress and acts as an osmoprotectant to reduce the negative effects of stress (Dong and Beckles, 2019), which might also be the reason why the starch content in grains of highland barley decreased under MP stress. We found that the total protein content was lower than the average content of highland barley, which could be related to the soil properties and climate. In terms of climatic conditions, the decrease in the monthly mean temperature and the number of sunshine hours were unfavorable for an increase in the protein content (Wang et al., 2017). For mineral elements, MP stress significantly reduced the content of Zn but had no significant effect on other nutrient elements (*P* < 0.05). Research indicates a correlation between highland barley's mineral composition and the soil nutrient levels (Birsin et al., 2010). Further analysis indicated that Fe, Zn, K, Mn, and P of green barley correlated with soil constituents, unlike Ca, Cu, and Mg, which showed limited correlation (Zhang et al., 2021b). In the present study, no significant changes in most of the mineral elements were observed as a result of the MP's stress. To elucidate the potential mechanisms driving these observations, we propose the following explanations based on the physicochemical properties of MPs and the functional roles of microbiota (Yao et al., 2023). From a mechanistic perspective, microplastics themselves can act as a novel physical interface, whose surfaces can adsorb or desorb specific nutrient ions. Simultaneously, the input of MPs alters soil aggregate structure, porosity, and water movement, affecting the diffusion of nutrients in the soil solution and root contact opportunities. More importantly, as an exogenous source of organic carbon, MPs can stimulate the activity of indigenous microorganisms, disrupting the balance of carbon and nitrogen cycles. This perturbation may preferentially affect the transformation and fixation/release processes of trace elements that are closely coupled with the nitrogen cycle, and reprogram carbon source allocation (Lan et al., 2025). This may explain why carbon-based reserve substances such as starch are degraded into soluble sugars to maintain osmotic balance under MP stress, leading to a decrease in their content.

The introduction of the MPDSM actively intervenes in this process through a “metabolic complementarity” effect (Shao et al., 2025). Its constituent strains may directly secrete organic acids, siderophores, or specific chelating molecules, acidifying the rhizosphere microenvironment or directly dissolving insoluble phosphates, carbonates, and metal oxides in the soil, thereby mobilizing fixed elements such as Ca, Cu, and Zn. Furthermore, MPDSM may induce systemic resistance in plants, regulating root physiology and the composition of root exudates, indirectly creating a rhizosphere environment more conducive to nutrient activation and absorption (Chen et al., 2025). Their metabolic activities may also directly or indirectly participate in the anabolic pathways of proteins, fats, and vitamins in plants. This study found that the inoculation with the MPDSM treatment group significantly increased the content of total proteins, total sugars, total flavonoids, and total polyphenols in the grains. This is consistent with the finding of Begum et al. (2022) that inoculation of bacteria under drought stress significantly promoted the accumulation of phenolics and flavonoids in tobacco (*Nicotiana tabacum* L.) (Begum et al., 2022). Furthermore, a large number of studies have also reported that inoculation with bacteria can improve plant yield and nutritional supplementation (Naveed et al., 2020; Scudeletti et al., 2021). The interaction between plants and microbes is considered to be the main factor leading to changes in the plant's nutrient composition. Microbes can convert nutrients that are not available in the soil into a usable form for plants to utilize (Das et al., 2022). Microbial metabolites may also be taken up by the roots of plants to promote their growth. Moreover, root exudates promote the proliferation of microbial, notably PGPR. This symbiotic alliance solidifies the plant-microbial ties (Upadhyay et al., 2022). In comparison to the large particle size MP treatment group, the inoculation with the MPDSM treatment could significantly increase the contents of fat, total polyphenols, and vitamin C; in comparison to the small particle size MP treatment group, the inoculation with the MPDSM treatment could significantly increase the contents of fat, total sugars, β-glucans, and vitamin C. This signifies that inoculation with the MPDSM remarkably enhances plant nutrition, benefitting them even under MP stress conditions. Begum et al. (2022) observed the same phenomenon in tobacco grown in drought environments (Begum et al., 2022). Inoculation of artificial synthetic flora has been used to address the negative effects of drought on plants (Begum et al., 2022; Santana et al., 2020), but the effect on plants under MP stress is still unknown. This study reported the changes in the nutrient content of highland barley grains under the treatments of MPs and the inoculation of the MPDSM that helps to improve our understanding of the symbiotic network and the compound ecological effects between plants and microbes under MP stress.

### 4.3 Fungal diversity and community structure

Microbes in the rhizosphere provide crops resistance against pathogens and abiotic stress (Alonso-Monge et al., 2009). Rhizosphere microbes enhance plant mineral absorption, influencing crop growth (Berendsen et al., 2012; Trivedi et al., 2020). As one of the most abundant taxa in soil, fungi play a critical role in the soil ecosystem processes (Egidi et al., 2019; Hamayun et al., 2009). Through analyzing the fungal diversity and community structure of the rhizosphere soil, we found that, except for the large-particle size B10 sample, the Simpson, Shannon, observed species, Chao1, and Pielou_e indices of the fungal communities in all samples increased. The findings indicate that the combined influence of MPs and the introduction of the MPDSM significantly impacted the rhizosphere fungal biodiversity. Our previous study found that inoculating the MPDSM reduced bacterial diversity and richness (Xiang et al., 2023a). However, inoculation with the MPDSM in this study increased the diversity and richness of the fungal community in the soil. Therefore, we considered that inoculation with the MPDSM only caused competition among bacteria in the soil but would not affect the competition between the fungal communities in the soil. In contrast, inoculation with the MPDSM could also promote synergy between the fungal communities in the soil, increasing both fungal diversity and abundance. Some studies have found that MPs at different concentrations can change the soil fungal community, with more sensitivity than the bacterial community; this is consistent with our results (Li et al., 2023; Fan et al., 2022). Crop rhizosphere fungal community structure alterations impinge on the crop's environmental adaptability, expansion, maturity, productivity, and quality, echoing our findings regarding the highland barley's nutritional stature (Li et al., 2022a). Furthermore, in addition, MPs and inoculation with the MPDSM treatments altered the structure of the fungal community in the crop rhizosphere. Statistical analysis at the genus level revealed that the MP treatment significantly increased the abundance of *Blumeria* and significantly decreased the abundance of *Verticillium* and *Gibberlla* in the rhizosphere soil. This may be related to strong environmental tolerance and ecological effects. Studies have reported that *Blumeria* is a pathogen in many crops; it can cause powdery mildew in graminaceous plants, affect plant growth, and reduce the quality of the plants (Dean et al., 2012; Mapuranga et al., 2022). The inoculation with the MPDSM treatment significantly increased the abundance of *Blumeria* and *Pleurotus* and decreased the abundance of *Botryotrichum, Verticillium*, and *Gibberella*. The enrichment of Blumeria in inter-root soils of MP- and MPDSM-treated barley increased the risk of crop pathogenicity. Research has found that *Pleurotus* can promote the growth of plants (Pham et al., 2019). *Botryotrichum* can secrete mycotoxins, which are toxic to cells (Rajachan et al., 2017), and a decrease in its abundance is beneficial for plant growth. In general, inoculation with the MPDSM treatment increased the abundance of fungal genera that are conducive to plant growth and improved the grain nutritional quality in the rhizosphere soil, reflecting that inoculation with the MPDSM treatment has a good effect on regulating soil microbiota and promoting plant growth. This research details changes to the rhizosphere soil's fungal community due to MP's intervention and the application of the MPDSM. In the remediation process of contaminated soil, the selection of microorganisms that can stably colonize the rhizosphere of crops and promote their growth is the focus of future research.

### 4.4 Fungal community functional prediction

We employed FUNGuild to analyze the impact of MP's and inoculation of the MPDSM on fungal functions. The results indicated that the function of the rhizosphere fungal community in the soil treated with MPs and inoculated with the MPDSM differentiated. Compared with the control, the B1 sample was enriched in the functions of Dung_Saprotroph-Ectomycorrhizal, Undefined_Saprotroph, and Animal_Pathogen-Clavicipitaceous_Endophyte-Saprotrophy; the B10 sample was enriched in the Wood_Saprotroph function; and the B50 sample was enriched in the Endophyte-Plant_Pathogen-Wood_Saprotroph function. The functions of Ectomycorrhizal and Animal_Pathogen in the S1 sample, the function of Dung_Saprotroph-Endophyte-Wood_Saprotroph in the S10 samples, and the function of Plant_Pathogen-Wood_Saprotroph in the S50 samples were higher than those in the other treatment groups. The inoculated group's SA1 samples had notably amplified Undefined Saprotroph-Undefined Biotroph and Fungal Parasite-Plant Pathogen activities compared to others. Some studies have found that the Wood_Saprotroph, Animal_Pathogen, and Plant_Pathogen-Wood_Saprotroph functional groups are changed by environmental pressure (Sen et al., 2021). The treatments of MPs with varying concentrations and particle sizes and the inoculation of the MPDSM resulted in the differentiation of functional groups of fungi in the soil rhizosphere. The enrichment of fungal functional groups reflected the response measures of soil fungi under environmental pressure.

### 4.5 Correlation analysis between nutrition and fungal diversity

The microbial rhizosphere community is significant for plant vitality, productivity, nutrient balance, and tolerance against biotic and abiotic stresses (Granzow et al., 2017; Middleton et al., 2021; Ma et al., 2021). In this study, Pearson correlation analysis showed that the nutritional indices of highland barley treated with different concentrations and particle sizes of MPs and MPDSM were correlated with the fungal biodiversity in the rhizosphere soil. Of particular note, at the genus level, *Gibberella* was significantly positively correlated with Cu, Mg, and Zn, *Humicola* with K, *Ampelomyces* with Mn, and *Cladosporium* with total sugar content. Only the *Conocybe* was significantly negatively correlated with the K, Cu, and Mg contents (*P* < 0.05). Liu et al. (2022) found that there was a significant correlation between the growth conditions and nutrient content of barley (*Hordeum vulgare*) and the composition of its microbial community. Soil pH and organic matter primarily govern the microbial composition in the rhizosphere soil during varied growth scenarios (Li et al., 2022c). In this paper, treatments with different concentrations and particle sizes of MPs and inoculation with the MPDSM treatment resulted in changes in soil organic matter, which, in turn, affected changes in soil microbial diversity and ultimately led to differences in plant nutrient content. Here, we analyzed the relationship between the rhizosphere fungal diversity and crop nutrition under MP stress and MPDSM treatment. This improves readers' understanding of the relationship between crop nutrition and microorganisms and provides a reference for future researchers to improve crop nutrition by using synthetic flora.

## 5 Conclusions

This study employed Highland barley as the experimental subject to investigate the effects of MP and inoculation with the MPDSM on grain nutrition and the structure and function of the fungal community in the rhizosphere soil through field experiments. The results showed that, after inoculation with MPDSM, the weight loss rate of large MPs (19.9%) was higher than that of small MPs (7.4%). However, SEM images showed that small MPs had a higher degree of degradation. MP treatment with a large particle size significantly increased the total flavonoid content of barley but decreased the contents of Ca, Zn, and Na. The small particle size MP treatment significantly increased the content of total polyphenols and vitamin E in highland barley and decreased the content of Zn (*P* < 0.05). The MPDSM treatment could improve the nutritional indexes of highland barley, including fat and vitamin C. Interestingly, the α-diversity (including the Shannon, Simpson, Chao 1, Observed Features, and Pielou_e indices) index was improved in all treatment groups compared to the control, except for the large particle size, medium concentration (1–5 mm, 10 g/m^2^) MPs treatment. Inoculation with the MPDSM increased the diversity of fungi in the rhizosphere and led to the differentiation of fungal communities. FUNGuild found that the treatments of MPs and inoculation of the MPDSM treatments led to the enrichment of Fungal_Parasite-Undefined_Saprotroph, Animal_Pathogen-Endophyte-Plant_Pathogen-Wood_Saprotroph, and Ectomycorrhizal functions. Soil fungal communities contaminated with MPs of various sizes and concentrations exhibit unique functions in mitigating adverse environmental impacts. The present study reveals the effects of MPs and the inoculation of the MPDSM on crop nutrients and rhizosphere fungi, which are crucial for understanding the complex ecological significance and bioremediation of MPs. The results of this study serve as the foundation for future research on the practical application of synthetic flora in field trials. In addition, we also propose that, in future research, a class of functional microorganisms that can not only remediate MP-contaminated soil but also promote the growth of crops should be further explored.

## Data Availability

The data presented in the study are deposited in the NCBI repository, accession number PRJNA1373447.

## Supplementary material

Supplementary Figure S1 Sparse curves of bacterial OTUs in different samples. CK, MPs-free, uninoculated microplastic-degrading synthetic microbiota (MPDSM) soil; B1, large particle size, low concentration soil (1–5 mm, 1 g/m^2^); B10, large particle size, medium concentration soil (1–5 mm, 10 g/m^2^); B50, large particle size, high concentration soil (1–5 mm, 50 g/m^2^); S1, small particle size, low concentration soil (< 1 mm, 1 g/m^2^); S10, small particle size, medium concentration soil (< 1 mm, 10 g/m^2^); S50, small particle size, high concentration soil (< 1 mm, 50 g/m^2^); CKA1, only inoculated MPDSM soil; BA1, large particle size, high concentration (1–5 mm, 50 g/m^2^), inoculated MPDSM soil; SA1, small particle size, high concentration (< 1 mm, 50 g/m^2^), inoculated MPDSM soil.

Supplementary Figure S2 Unique and common operational taxonomic units (OTUs) between different samples. CK, MPs-free, uninoculated microplastic-degrading synthetic microbiota (MPDSM) soil; B1, large particle size, low concentration soil (1–5 mm, 1 g/m^2^); B10, large particle size, medium concentration soil (1–5 mm, 10 g/m^2^); B50, large particle size, high concentration soil (1–5 mm, 50 g/m^2^); S1, small particle size, low concentration soil (< 1 mm, 1 g/m^2^); S10, small particle size, medium concentration soil (< 1 mm, 10 g/m^2^); S50, small particle size, high concentration soil (< 1 mm, 50 g/m^2^); CKA1, only inoculated MPDSM soil; BA1, large particle size, high concentration (1–5 mm, 50 g/m^2^), inoculated MPDSM soil; SA1, small particle size, high concentration (< 1 mm, 50 g/m^2^), inoculated MPDSM soil.

Supplementary Figure S3 Differential analysis of fungal taxa in different samples based on principal component analysis (PCA). CK, MPs-free, uninoculated microplastic-degrading synthetic microbiota (MPDSM) soil; B1, large particle size, low concentration soil (1–5 mm, 1 g/m^2^); B10, large particle size, medium concentration soil (1–5 mm, 10 g/m^2^); B50, large particle size, high concentration soil (1–5 mm, 50 g/m^2^); S1, small particle size, low concentration soil (< 1 mm, 1 g/m^2^); S10, small particle size, medium concentration soil (< 1 mm, 10 g/m^2^); S50, small particle size, high concentration soil (< 1 mm, 50 g/m^2^); CKA1, only inoculated MPDSM soil; BA1, large particle size, high concentration (1–5 mm, 50 g/m^2^), inoculated MPDSM soil; SA1, small particle size, high concentration (< 1 mm, 50 g/m^2^), inoculated MPDSM soil.

## References

[B1] Almeida A. Loy A. Hofmann H. (2018). ggplot2 Compatible quantile-quantile plots in R. R J. 10, 248–261. doi: 10.32614/RJ-2018-051

[B2] Alonso-Monge R. Román E. Arana D. M. Pla J. Nombela C. (2009). Fungi sensing environmental stress. Clin. Microbiol. Infect. 15, 17–9. doi: 10.1111/j.1469-0691.2008.02690.x 19220347

[B3] Araya T. Nyssen J. A. N. Govaerts B. Baudron F. Carpentier L. Bauer H. . (2016). Restoring cropland productivity and profitability in northern ethiopian drylands after nine years of resource-conserving agriculture. Exp. Agric. 52, 165–187. doi: 10.1017/S001447971400060X

[B4] Begum N. Wang L. Ahmad H. Akhtar K. Roy R. Khan M. I. . (2022). Co-inoculation of arbuscular mycorrhizal fungi and the plant growth-promoting rhizobacteria improve growth and photosynthesis in tobacco under drought stress by up-regulating antioxidant and mineral nutrition metabolism. Microb. Ecol. 83, 971–988. doi: 10.1007/s00248-021-01815-7 34309697

[B5] Berendsen R. L. Pieterse C. M. J. Bakker P. A. H. M. (2012). The rhizosphere microbiome and plant health. Trends Plant Sci. 17, 478–486. doi: 10.1016/j.tplants.2012.04.001 22564542

[B6] Birsin M. A. Adak M. S. Inal A. Aksu A. Gunes A. (2010). Mineral element distribution and accumulation patterns within two barley cultivars. J. Plant Nutr. 33, 267–284. doi: 10.1080/01904160903435391

[B7] Brodhagen M. Goldberger J. R. Hayes D. G. Inglis D. A. Marsh T. L. Miles C. . (2017). Policy considerations for limiting unintended residual plastic in agricultural soils. Environ. Sci. Policy 69, 81–84. doi: 10.1016/j.envsci.2016.12.014

[B8] Caporaso J. G. Kuczynski J. Stombaugh J. Bittinger K. Bushman F. D. Costello E. K. . (2010). QIIME allows analysis of high-throughput community sequencing data. Nat. Methods 7, 335–6. doi: 10.1038/nmeth.f.303 20383131 PMC3156573

[B9] Chang Y. Lv Y. (2016). Structure, functionality and digestibility of acetylated hulless barley starch. Int. J. Food Prop. 20, 1818–1828. doi: 10.1080/10942912.2016.1220013

[B10] Chen S. Liu Q. Li D. (2025). Synthetic microbial community enhances lignocellulose degradation at the composting thermophilic phase: metagenomic and metabolic pathway insights. Chem. Eng. J. 520:165847. doi: 10.1016/j.cej.2025.165847

[B11] Das P. P. Singh K. R. Nagpure G. Mansoori A. Singh R. P. Ghazi I. A. . (2022). Plant-soil-microbes: a tripartite interaction for nutrient acquisition and better plant growth for sustainable agricultural practices. Environ. Res. 214:113821. doi: 10.1016/j.envres.2022.113821 35810815

[B12] De Souza Machado A. A. Lau C. W. Kloas W. Bergmann J. Bachelier J. B. Faltin A. S. . (2019). Microplastics can change soil properties and affect plant performance. Environ. Sci. Technol. 53, 6044–6052. doi: 10.1021/acs.est.9b01339 31021077

[B13] Dean R. Van Kan J. A. Pretorius Z. A. Hammond-Kosack K. E. Di Pietro A. Spanu J. . (2012). The Top 10 fungal pathogens in molecular plant pathology. Mol. Plant Pathol. 13, 414–30. doi: 10.1111/j.1364-3703.2011.00783.x 22471698 PMC6638784

[B14] Do T. D. T. Cozzolino D. Muhlhausler B. Box A. Able A. J. (2015). Antioxidant capacity and vitamin E in barley: effect of genotype and storage. Food Chem. 187, 65–74. doi: 10.1016/j.foodchem.2015.04.028 25976999

[B15] Dong S. Beckles D. M. (2019). Dynamic changes in the starch-sugar interconversion within plant source and sink tissues promote a better abiotic stress response. J. Plant Physiol. 234–235, 80–93. doi: 10.1016/j.jplph.2019.01.007 30685652

[B16] Du H. Wang J. (2021). Characterization and environmental impacts of microplastics. Gondwana Res. 98, 63–75. doi: 10.1016/j.gr.2021.05.023

[B17] Edgar R. C. (2004). MUSCLE: multiple sequence alignment with high accuracy and high throughput. Nucleic Acids Res. 32, 1792–7. doi: 10.1093/nar/gkh340 15034147 PMC390337

[B18] Edgar R. C. (2013). UPARSE: highly accurate OTU sequences from microbial amplicon reads. Nat. Methods 10, 996–998. doi: 10.1038/nmeth.2604 23955772

[B19] Egidi E. Delgado-Baquerizo M. Plett J. M. Wang J. Eldridge D. J. Bardgett R. D. . (2019). A few ascomycota taxa dominate soil fungal communities worldwide. Nat. Commun. 10:2369. doi: 10.1038/s41467-019-10373-z 31147554 PMC6542806

[B20] Fan P. Tan W. B. Yu H. (2022). Effects of different concentrations and types of microplastics on bacteria and fungi in alkaline soil. Ecotoxicol. Environ. Saf. 229:113045. doi: 10.1016/j.ecoenv.2021.113045 34890986

[B21] Gao H. Yan C. Liu Q. Ding W. Chen B. Li Z. . (2019). Effects of plastic mulching and plastic residue on agricultural production: a meta-analysis. Sci. Total Env. 651, 484–492. doi: 10.1016/j.scitotenv.2018.09.105 30243168

[B22] García-Depraect O. Lebrero R. Rodriguez-Vega S. Bordel S. Santos-Beneit F. Martínez-Mendoza L. J. . (2022). Biodegradation of bioplastics under aerobic and anaerobic aqueous conditions: kinetics, carbon fate and particle size effect. Bioresour. Technol. 344:126265. doi: 10.1016/j.biortech.2021.126265 34737051

[B23] Granzow S. Kaiser K. Wemheuer B. Pfeiffer B. Daniel R. Vidal S. . (2017). The effects of cropping regimes on fungal and bacterial communities of wheat and faba bean in a greenhouse pot experiment differ between plant species and compartment. Front. Microbiol. 8:902. doi: 10.3389/fmicb.2017.00902 28611735 PMC5447230

[B24] Guo T. Horvath C. Chen L. Chen J. Zheng B. (2020). Understanding the nutrient composition and nutritional functions of highland barley (Qingke): a review. Trends Food Sci. Technol. 103, 109–117. doi: 10.1016/j.tifs.2020.07.011

[B25] Hamayun M. Khan S. A. Iqbal I. Na C. I. Khan A. L. Hwang Y. H. . (2009). Chrysosporium pseudomerdarium produces gibberellins and promotes plant growth. J. Microbiol. 47, 425–30. doi: 10.1007/s12275-009-0268-6 19763416

[B26] Horton A. A. Walton A. Spurgeon D. J. Lahive E. Svendsen C. (2017). Microplastics in freshwater and terrestrial environments: evaluating the current understanding to identify the knowledge gaps and future research priorities. Sci. Total Env. 586, 127–141. doi: 10.1016/j.scitotenv.2017.01.190 28169032

[B27] Huang F. L. Bao C. G. Peng M. Zhu G. L. He Z. B. Chen X. F. . (2015). Chromatographic analysis of fatty acid composition in differently sized seeds of castor accessions. Biotechnol. Biotechnol. Equip. 29, 892–900. doi: 10.1080/13102818.2015.1053410

[B28] Iqbal S. Li Y. Xu J. Worthy F. R. Gui H. Faraj T. K. . (2025). Smallest microplastics intensify maize yield decline, soil processes and consequent global warming potential. J. Hazard. Mater. 486:136993. doi: 10.1016/j.jhazmat.2024.136993 39754884

[B29] Keswani C. Singh S. P. García-Estrada C. Mezaache-Aichour S. Glare T. R. Borriss R. . (2022). Biosynthesis and beneficial effects of microbial gibberellins on crops for sustainable agriculture. J. Appl. Microbiol. 132, 1597–1615. doi: 10.1111/jam.15348 34724298

[B30] Khatri D. Chhetri S. B. B. (2020). Reducing sugar, total phenolic content, and antioxidant potential of nepalese plants. Biomed Res. Int. 2020:7296859. doi: 10.1155/2020/7296859 33274222 PMC7683130

[B31] Lan G. Huang X. Li T. Huang Y. Liao Y. Zheng Q. . (2025). Effect of microplastics on carbon, nitrogen and phosphorus cycle in farmland soil: a meta-analysis. Environ. Pollut. 370:125871. doi: 10.1016/j.envpol.2025.125871 39971082

[B32] Leifheit E. F. Lehmann A. Rillig M. C. (2021). Potential effects of microplastic on arbuscular mycorrhizal fungi. Front. Plant Sci. 12:626709. doi: 10.3389/fpls.2021.626709 33597964 PMC7882630

[B33] Li K. Zhang M. J. Jia W. Q. Xu L. B. Huang Y. (2023). Deciphering the effects of LDPE microplastic films on diversity, composition and co-occurrence network of soil fungal community. Appl. Soil Ecol. 182:104716. doi: 10.1016/j.apsoil.2022.104716

[B34] Li Q. Wu Q. Zhang T. Xiang P. Bao Z. Tu W. . (2022a). Phosphate mining activities affect crop rhizosphere fungal communities. Sci. Total Env. 838:156196. doi: 10.1016/j.scitotenv.2022.156196 35623536

[B35] Li Q. Xiang P. Zhang T. Wu Q. Bao Z. Tu W. . (2022b). The effect of phosphate mining activities on rhizosphere bacterial communities of surrounding vegetables and crops. Sci. Total Environ. 821:153479. doi: 10.1016/j.scitotenv.2022.153479 35092784

[B36] Li W. Lei X. Zhang R. Cao Q. Yang H. Zhang N. . (2022c). Shifts in rhizosphere microbial communities in Oplopanax elatus Nakai are related to soil chemical properties under different growth conditions. Sci. Rep. 12:11485. doi: 10.1038/s41598-022-15340-1 35798802 PMC9262954

[B37] Lin S. Guo H. Gong J. D. B. Lu M. Lu M-. Y. Wang L. . (2018). Phenolic profiles, β-glucan contents, and antioxidant capacities of colored Qingke (Tibetan hulless barley) cultivars. J. Cereal Sci. 81, 69–75. doi: 10.1016/j.jcs.2018.04.001

[B38] Liu J. Wang Z. Chen Z. White J. F. Malik K. Chen T. . (2022). Inoculation of barley (*Hordeum vulgare*) with the endophyte *Epichloë bromicola* affects plant growth, and the microbial community in roots and rhizosphere soil. J. Fungi 8:172. doi: 10.3390/jof8020172 35205925 PMC8876963

[B39] Liu M. Lu S. Song Y. Lei L. Hu J. Lv W. . (2018). Microplastic and mesoplastic pollution in farmland soils in suburbs of Shanghai, China. Environ. Pollut. 242, 855–862. doi: 10.1016/j.envpol.2018.07.051 30036839

[B40] Ma S. Lin Y. Qin Y. Diao X. Li P. (2021). Microbial diversity characteristics of areca palm rhizosphere soil at different growth stages. Plants 10:2706. doi: 10.3390/plants10122706 34961178 PMC8705836

[B41] Magoc T. Salzberg S. L. (2011). FLASH: fast length adjustment of short reads to improve genome assemblies. Bioinformatics 27, 2957–2963. doi: 10.1093/bioinformatics/btr507 21903629 PMC3198573

[B42] Mapuranga J. Chang J. Yang W. (2022). Combating powdery mildew: advances in molecular interactions between Blumeria graminis f. sp. tritici and wheat. Front. Plant Sci. 13:1102908. doi: 10.3389/fpls.2022.1102908 36589137 PMC9800938

[B43] Middleton H. Yergeau É. Monard C. Combier J. P. El Amrani A. (2021). Rhizospheric plant-microbe interactions: miRNAs as a key mediator. Trends Plant Sci. 26, 132–141. doi: 10.1016/j.tplants.2020.09.005 33036916

[B44] Naveed M. Mustafa A. Majeed S. Naseem Z. Saeed Q. Khan A. . (2020). Enhancing cadmium tolerance and pea plant health through *Enterobacter* sp. MN17 inoculation together with biochar and gravel sand. Plants 9:530. doi: 10.3390/plants9040530 32326023 PMC7238170

[B45] Nguyen N. H. Song Z. W. Bates S. T. Branco S. Tedersoo L. Menke J. . (2016). FUNGuild: an open annotation tool for parsing fungal community datasets by ecological guild. Fungal Ecol. 20, 241–248. doi: 10.1016/j.funeco.2015.06.006

[B46] Nilsson R. H. Larsson K. H. Taylor A. F. S. Bengtsson-Palme J. Jeppesen T. S. Schigel D. . (2019). The UNITE database for molecular identification of fungi: handling dark taxa and parallel taxonomic classifications. Nucleic Acids Res. 47, D259–D264. doi: 10.1093/nar/gky1022 30371820 PMC6324048

[B47] Pham M. T. Huang C. M. Kirschner R. (2019). The plant growth-promoting potential of the mesophilic wood-rot mushroom *Pleurotus pulmonarius*. J. Appl. Microbiol. 127, 1157–1171. doi: 10.1111/jam.14375 31291682

[B48] Quast C. Pruesse E. Yilmaz P. Gerken J. Schweer T. Yarza P. . (2013). The SILVA ribosomal RNA gene database project: improved data processing and web-based tools. Nucleic Acids Res. 41, D590–D596. doi: 10.1093/nar/gks1219 23193283 PMC3531112

[B49] Rajachan O. A. Kanokmedhakul K. Soytong K. Kanokmedhakul S. (2017). Mycotoxins from the Fungus *Botryotrichum piluliferum*. J. Agric. Food Chem. 65, 1337–1341. doi: 10.1021/acs.jafc.6b05522 28135416

[B50] Roager L. Sonnenschein E. C. (2019). Bacterial candidates for colonization and degradation of marine plastic debris. Environ. Sci. Technol. 53, 11636–11643. doi: 10.1021/acs.est.9b02212 31557003

[B51] Rousk J. Bååth E. Brookes P. C. Lauber C. L. Lozupone C. Caporaso J. G. . (2010). Soil bacterial and fungal communities across a pH gradient in an arable soil. ISME J. 4, 1340–1351. doi: 10.1038/ismej.2010.58 20445636

[B52] Santana S. R. A. Voltolini T. V. Antunes G. D. R. Da Silva V. M. Simões W. L. Morgante S. T. . (2020). Inoculation of plant growth-promoting bacteria attenuates the negative effects of drought on sorghum. Arch. Microbiol. 202, 1015–1024. doi: 10.1007/s00203-020-01810-5 31932864

[B53] Scudeletti D. Crusciol C. A. C. Bossolani J. W. Moretti L. G. Momesso L. Servaz Tubaña B. . (2021). *Trichoderma asperellum* inoculation as a tool for attenuating drought stress in sugarcane. Front. Plant Sci. 12:645542. doi: 10.3389/fpls.2021.645542 33936132 PMC8082249

[B54] Sen K. Bai M. Sen B. Wang G. (2021). Disentangling the structure and function of mycoplankton communities in the context of marine environmental heterogeneity. Sci. Total Environ. 766:142635. doi: 10.1016/j.scitotenv.2020.142635 33071110

[B55] Shah A. Smith D. (2020). Flavonoids in agriculture: chemistry and roles in, biotic and abiotic stress responses, and microbial associations. Agronomy 10:1209. doi: 10.3390/agronomy10081209

[B56] Shao Y. Gu S. Peng H. Zhang L. Li S. Berendsen R. L. . (2025). Synergic interactions between Trichoderma and the soil microbiomes improve plant iron availability and growth. NPJ Biofilms Microbiomes 11:56. doi: 10.1038/s41522-025-00684-z 40199867 PMC11978894

[B57] Sharma A. Shahzad B. Rehman A. Bhardwaj R. Landi M. Zheng B. . (2019). Response of phenylpropanoid pathway and the role of polyphenols in plants under abiotic stress. Molecules 24:2452. doi: 10.3390/molecules24132452 31277395 PMC6651195

[B58] Shirin J. Chen Y. Hussain Shah A. Da Y. Zhou G. Sun Q. . (2024). Micro plastic driving changes in the soil microbes and lettuce growth under the influence of heavy metals contaminated soil. Front Plant Sci. 15:2024. doi: 10.3389/fpls.2024.1427166 39323532 PMC11422782

[B59] Steinmetz Z. Wollmann C. Schaefer M. Buchmann C. David J. Tröger J. . (2016). Plastic mulching in agriculture. Trading short-term agronomic benefits for long-term soil degradation? Sci. Total Env. 550, 690–705. doi: 10.1016/j.scitotenv.2016.01.153 26849333

[B60] Temporiti M. E. E. Nicola L. Girometta C. E. Roversi A. Daccò C. Tosi S. . (2022). The analysis of the mycobiota in plastic polluted soil reveals a reduction in metabolic ability. J. Fungi 8:1247. doi: 10.3390/jof8121247 36547580 PMC9785340

[B61] Teslová P. Kalina J. Urban O. (2010). Simultaneous determination of non-structural saccharides and starch in leaves of higher plants using anthrone reagent. Chemicke Listy 104, 867–870.

[B62] Trivedi P. Leach J. E. Tringe S. G. Sa T. Singh B. K. (2020). Plant–microbiome interactions: from community assembly to plant health. Nat. Rev. Microbiol. 18, 607–621. doi: 10.1038/s41579-020-0412-1 32788714

[B63] Upadhyay S. K. Srivastava A. K. Rajput V. D. Chauhan P. K. Bhojiya A. A. Jain D. . (2022). Root exudates: mechanistic insight of plant growth promoting rhizobacteria for sustainable crop production. Front. Microbiol. 13:916488. doi: 10.3389/fmicb.2022.916488 35910633 PMC9329127

[B64] Wang J. Zhiming Z. Xibo F. Gang F. Weihai H. Gaihua W. . (2017). Regulation of protein content of naked barley varieties and its relationships with environmental factors in Qinghai-Tibet plateau. Scientia Agricultura Sinica 50, 969–977.

[B65] Xiang P. Liao W. Xiong Z. Xiao W. Luo Y. Peng L. . (2023a). Effects of polystyrene microplastics on the agronomic traits and rhizosphere soil microbial community of highland barley. Sci. Total Environ. 907:167986. doi: 10.1016/j.scitotenv.2023.167986 37879483

[B66] Xiang P. Zhang T. Wu Q. Li Q. (2023b). Systematic review of degradation processes for microplastics: progress and prospects. Sustainability 15:12698. doi: 10.3390/su151712698

[B67] Xiang P. Zhang Y. Zhang T. Wu Q. Zhao C. Li Q. (2023c). A novel bacterial combination for efficient degradation of polystyrene microplastics. J. Hazard. Mater. 458:131856. doi: 10.1016/j.jhazmat.2023.131856 37331064

[B68] Yao S. Li X. Wang T. Jiang X. Song Y. Arp H. P. H. . (2023). Soil Metabolome impacts the formation of the eco-corona and adsorption processes on microplastic surfaces. Environ. Sci. Technol. 57, 8139–8148. doi: 10.1021/acs.est.3c01877 37194262 PMC10233752

[B69] Zhang T. Wang Q. Li J. Zhao S. Qie M. Wu X. . (2021a). Study on the origin traceability of Tibet highland barley (*Hordeum vulgare* L.) based on its nutrients and mineral elements. Food Chem. 346:128928. doi: 10.1016/j.foodchem.2020.128928 33412485

[B70] Zhang T. Wang Q. Li J. Zhao S. Qie M. Wu X. . (2021b). Study on the origin traceability of Tibet highland barley (*Hordeum vulgare* L.) based on its nutrients and mineral elements. Food Chem. 346:128928. 33412485 10.1016/j.foodchem.2020.128928

[B71] Zhu J. Liu S. Wang H. Wang D. Zhu Y. Wang J. . (2022). Microplastic particles alter wheat rhizosphere soil microbial community composition and function. J. Hazard. Mater. 436:129176. doi: 10.1016/j.jhazmat.2022.129176 35739711

