# Supplementary material for: Synthetic microbiota for microplastic degradation modulates rhizosphere fungal diversity and metabolic function in highland barley
Source: Front Microbiol. 2025 Dec 8;16:1711544. doi: 10.3389/fmicb.2025.1711544 (PMC12722916; doi:10.3389/fmicb.2025.1711544)
Supplement: Supplementary file 1 [file Data_Sheet_1.docx]

**Table S1. Methods used for the determination of nutritional components in highland barley grains.**

| Nutrient | Analytical Method / Principle | **Reference/** Reagent kit manufacturer |
| --- | --- | --- |
| Starch | Anthrone staining after acid hydrolysis | (Zhang et al., 2021a, Teslová et al., 2010) |
| Protein | BCA method under alkaline conditions | (Zhang et al., 2021a) |
| Fat | Soxhlet extraction with organic solvents | (Huang et al., 2015) |
| Total Flavonoids | Colorimetry with aluminum trichloride | (Zhang et al., 2021a). |
| Total Phenolics | Folin-Ciocalteu method | (Khatri and Chhetri, 2020) |
| β-Glucan | Enzymatic assay (Megazyme kit) | (Lin et al., 2018) |
| Vitamins C & E | Commercial kit (Jiancheng Bioengineering) | Nanjing Jiancheng Biotechnology（A009-1-1 and A008-1-1） |
| Total Sugar | Acid hydrolysis followed by DNS method | Suzhou Greeth Biotechnology（G0503F） |
| Mineral Elements | ICP-OES after acid digestion | (Li et al., 2022b) |
