# Supplementary figures and images for: Synthetic microbiota for microplastic degradation modulates rhizosphere fungal diversity and metabolic function in highland barley
Source: Front Microbiol. 2025 Dec 8;16:1711544. doi: 10.3389/fmicb.2025.1711544 (PMC12722916; doi:10.3389/fmicb.2025.1711544)

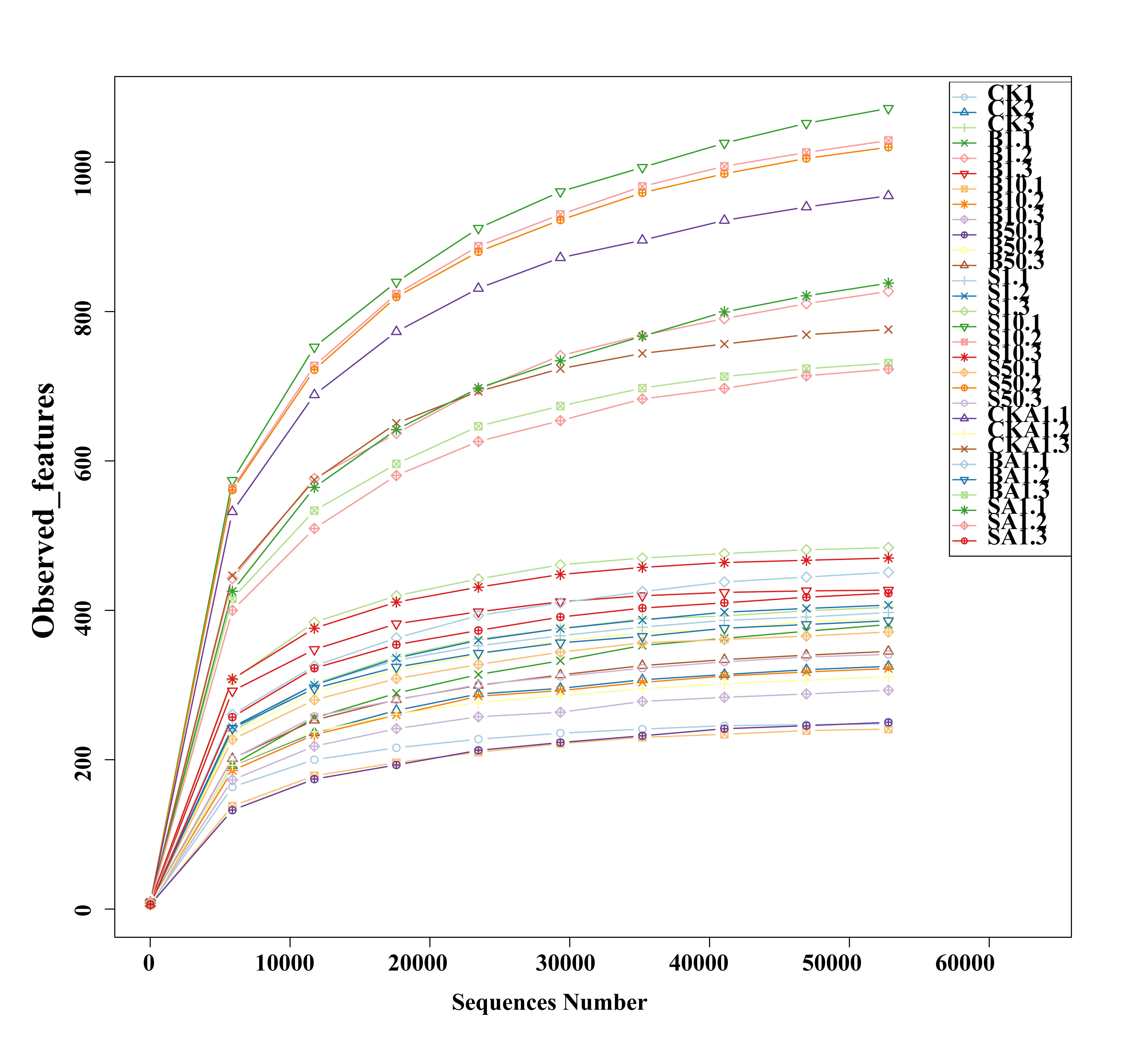

Supplement: Supplementary Figure S1 — Sparse curves of bacterial OTUs in different samples. CK, MPs-free, uninoculated microplastic-degrading synthetic microbiota (MPDSM) soil; B1, large particle size, low concentration soil (1–5 mm, 1 g/m2); B10, large particle size, medium concentration soil (1–5 mm, 10 g/m2); B50, large particle size, high concentration soil (1–5 mm, 50 g/m2); S1, small particle size, low concentration soil (< 1 mm, 1 g/m2); S10, small particle size, medium concentration soil (< 1 mm, 10 g/m2); S50, small particle size, high concentration soil (< 1 mm, 50 g/m2); CKA1, only inoculated MPDSM soil; BA1, large particle size, high concentration (1–5 mm, 50 g/m2), inoculated MPDSM soil; SA1, small particle size, high concentration (< 1 mm, 50 g/m2), inoculated MPDSM soil. [file Image_1.tif]

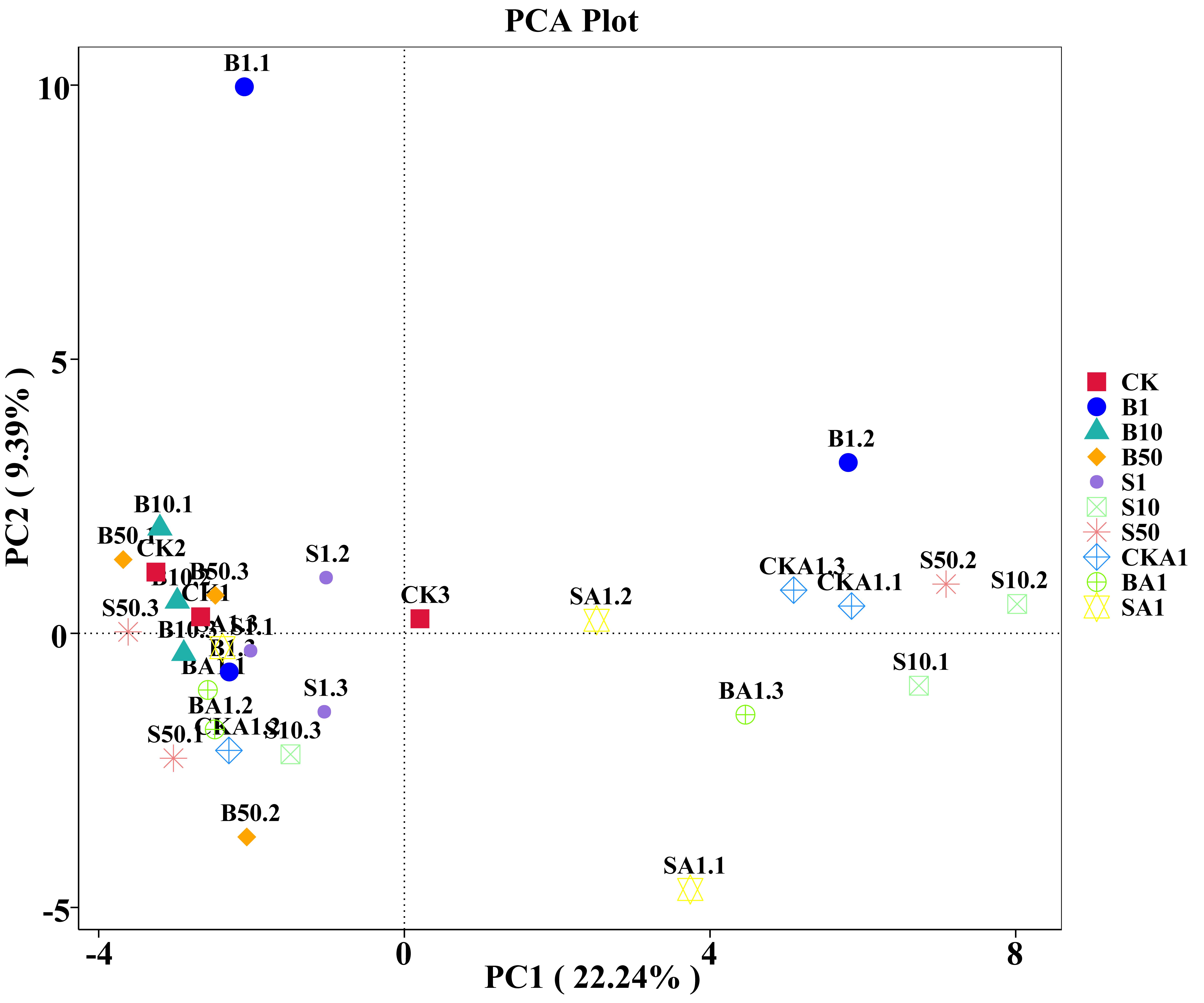

Supplement: Supplementary Figure S3 — Differential analysis of fungal taxa in different samples based on principal component analysis (PCA). CK, MPs-free, uninoculated microplastic-degrading synthetic microbiota (MPDSM) soil; B1, large particle size, low concentration soil (1–5 mm, 1 g/m2); B10, large particle size, medium concentration soil (1–5 mm, 10 g/m2); B50, large particle size, high concentration soil (1–5 mm, 50 g/m2); S1, small particle size, low concentration soil (< 1 mm, 1 g/m2); S10, small particle size, medium concentration soil (< 1 mm, 10 g/m2); S50, small particle size, high concentration soil (< 1 mm, 50 g/m2); CKA1, only inoculated MPDSM soil; BA1, large particle size, high concentration (1–5 mm, 50 g/m2), inoculated MPDSM soil; SA1, small particle size, high concentration (< 1 mm, 50 g/m2), inoculated MPDSM soil. [file Image_3.tif]
